# The biogeography of red snow microbiomes and their role in melting arctic glaciers

**DOI:** 10.1038/ncomms11968

**Published:** 2016-06-22

**Authors:** Stefanie Lutz, Alexandre M. Anesio, Rob Raiswell, Arwyn Edwards, Rob J. Newton, Fiona Gill, Liane G. Benning

**Affiliations:** 1 Cohen Laboratories, School of Earth and Environment, University of Leeds, Leeds LS2 9JT, UK; 2 GFZ German Research Centre for Geosciences, Telegrafenberg, Potsdam 14473, Germany; 3 Bristol Glaciology Centre, School of Geographical Sciences, University of Bristol, Bristol BS8 1SS, UK; 4 Institute of Biological, Environmental and Rural Sciences (IBERS), Aberystwyth University, Aberystwyth SY23 3FL, UK; 5 Interdisciplinary Centre for Environmental Microbiology, Aberystwyth University, Aberystwyth SY23 3FL, UK

## Abstract

The Arctic is melting at an unprecedented rate and key drivers are changes in snow and ice albedo. Here we show that red snow, a common algal habitat blooming after the onset of melting, plays a crucial role in decreasing albedo. Our data reveal that red pigmented snow algae are cosmopolitan as well as independent of location-specific geochemical and mineralogical factors. The patterns for snow algal diversity, pigmentation and, consequently albedo, are ubiquitous across the Arctic and the reduction in albedo accelerates snow melt and increases the time and area of exposed bare ice. We estimated that the overall decrease in snow albedo by red pigmented snow algal blooms over the course of one melt season can be 13%. This will invariably result in higher melt rates. We argue that such a ‘bio-albedo' effect has to be considered in climate models.

## Abstract (web summary)

The Arctic is melting at an unprecedented rate and key drivers are changes in snow and ice albedo. Here, the authors show that red pigmented snow algae play a crucial role in decreasing surface albedo and their patterns for diversity, pigmentation, and consequently albedo, are ubiquitous across the Arctic.

## Graphical abstract

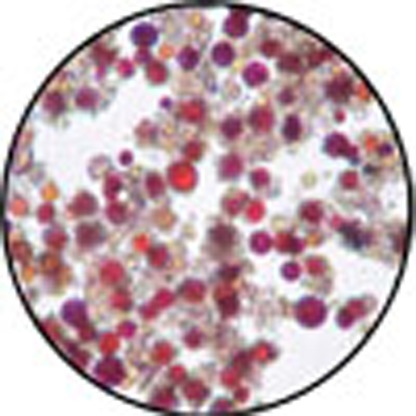

Glaciers are important components of Earth's climate and hydrologic system. The Arctic is being disproportionately affected by global warming, which in turn provides a strong feedback on the climate system^1^. One of the key parameters in the increase of glacial melt is albedo change^2^. The physical and chemical characteristics of snow and ice have been studied intensively; however, the field of glacial microbiology is still in its infancy. Snow and ice surfaces have been considered barren until recently, yet distinct habitats harbour species of all three domains of life^3^. So far, most attention has been paid to cryoconite holes^4^,^5^,^6^,^7^, which are dominated by bacteria^8^,^9^. These are, however, only active once the long-lasting snow cover has melted away, and their coverage on glaciated areas usually reaches a maximum of only 10% (refs 3, 4, 8). In contrast, little is known about the diversity or function of snow algae, nor their global effect on albedo and hence glacial melting. This is despite the fact that coloured snow algal blooms have been known since Aristotle^10^, and that they dominate primary production on snow and ice fields^11^,^12^.

For most of the year, the largest proportion of the glacial surfaces in the Arctic is covered by snow. Moreover, permanent and seasonal snow can cover up to 35% of the entire Earth's surface^13^. We have recently shown that snow algae are critical players in glacial surface habitats and the dominating biomass immediately after the onset of melting^11^. Snow algae are prolific primary colonizers and producers that can form extensive blooms in spring and summer. Such snow algal blooms can substantially darken the surface of glaciers because of their red pigmentation (secondary carotenoids), which the algae produce as a protection mechanism (for example, from high levels of irradiation)^14^,^15^. We have shown that this phenomenon, known as ‘red snow', can reduce the surface albedo locally by up to 20%, which in turn further increases melting rates of snow^11^. Previous studies have been unable to generalize this effect because of a lack of information on the distribution, and controls on red snow ecology and physiology. These studies have so far focussed on describing algae primarily through classical microbiological approaches^16^,^17^,^18^ (for example, microscopy). In contrast, in the current study, we have employed high-throughput sequencing to characterize these cryophilic micro-eukaryotes and their associated microbiota, that is, bacteria and archaea. We evaluated the diversity and functionality of the red snow algal habitat in four geographically well-separated glacial systems across the Arctic, comprising of 40 red snow sites on 16 glaciers and snow fields. This way, we have produced the first large-scale biogeographical data set for red pigmented snow algae. Knowledge of the global distribution of species and their underlying spatial patterns and processes (that is, their biogeography) has long been assumed irrelevant for microbial communities. However, recently documented rapid changes in diversity across many ecosystems have led to an increased focus on biogeographical patterns and traits in microorganisms^19^,^20^,^21^. Identifying patterns can help to better understand their ecology within a specific ecosystem and make predictions about their role on a larger scale. We cross-correlated the marker gene data with geochemical and metabolic measurements. These parameters were then used to evaluate the environmental forcing factors on the snow microbial community composition.

Furthermore, recent snow-albedo models for Greenland^22^ suggest that melting accelerates largely due to increased contributions from light-absorbing impurities, with impurities being primarily considered to be anthropogenic, forest fire-derived black carbon, Saharan or pro-glacial mineral dust^23^. However, the contribution of coloured algae to changing albedo and melt rates has not previously been considered^24^.

Here, based on our albedo measurements on red snow and comparing with literature data for algae-free snow, we have estimated the reduction of albedo caused by microbial darkening of glacial surfaces (inferring higher melting rates). This will help to improve our understanding of the response of glacial systems to a warming climate.

## Results

### Cosmopolitan algal but local bacterial community structure

**Figure 1:**
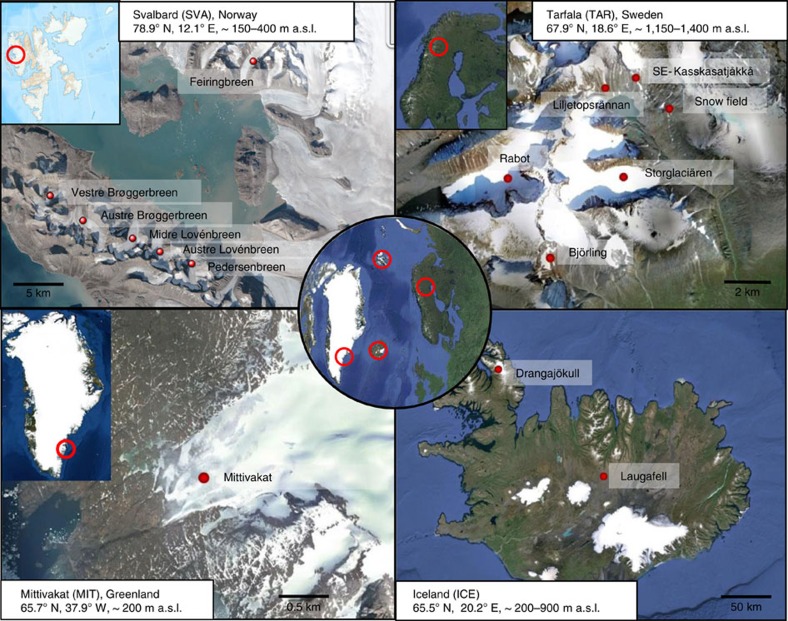
Sample locations. Locations of the 16 glaciers and snow fields across the Arctic, where 40 sites of red snow were sampled: Svalbard (*n*=12), Northern Sweden (*n*=24), Greenland (*n*=2) and Iceland (*n*=2). These localities were chosen as they represent different geographical settings including low (67.9°N) versus high (78.9°N) latitude, low (150–400 m) versus high (∼1,200–1,400 m) elevation, and maritime versus continental settings. Red dots represent sampling sites and several sampling events within one site (for full details, see Supplementary Table 1). Map data: Google, DigitalGlobe.

We have assessed the biogeographical patterns for red snow microbiomes across the Arctic by using high-throughput sequencing of the small subunit ribosomal RNA genes and characterized the species composition of 40 red snow sites in four well-separated and physico-chemically diverse Arctic settings (see Fig. 1 and Supplementary Table 1 for full details).

**Figure 2:**
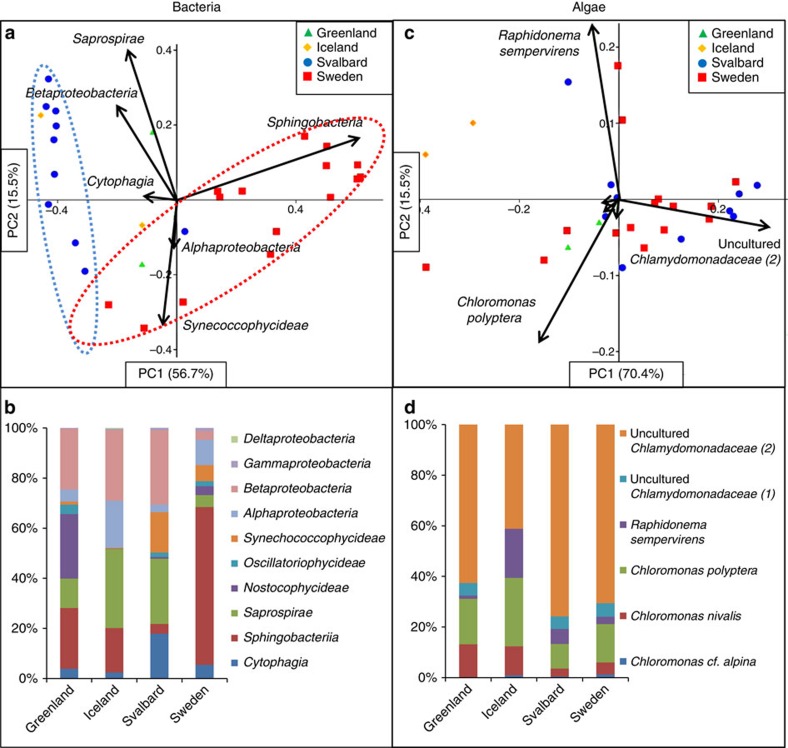
Algal and bacterial community composition. Principal component analysis of bacterial classes (**a**,**b**) and algal species (**c**,**d**) revealing taxonomic distance between sampling sites and taxa causing separation. Algal species show homogenous community composition across all sites (**c**), whereas bacteria cluster according to locations, even on the higher taxonomic class level (**a**, dotted lines have been added to help guide the reader's eye). Bar charts show average community composition (Supplementary Tables 2 and 4 for individual values; Supplementary Table 3 for averages and *P*-values) for each location and confirm similar composition for algae (**d**) but large differences for bacteria (**b**).

Our results show that, similar to recent studies of other habitats (for example, soil, marine)^25^,^26^,^27^, the bacteria in our red snow samples inherited a strong geographical separation, despite their small cell size and therefore high potential for universal distribution. Bacteria were mostly represented by the phyla *Bacteriodetes*, *Proteobacteria* and *Cyanobacteria* (Supplementary Table 2). These bacterial phyla have previously been described in snow environments^13^,^28^,^29^. However, we found significant differences (*P*<0.05) between locations for the major classes within these phyla (Supplementary Table 3). Samples clustered according to their geographic location (Fig. 2a), and the observed differences were derived from large variations in the relative abundance of *Sphingobacteria*, *Saprospirae*, *Alphaproteobacteria*, *Betaproteobacteria* and *Synechococcophycideae*. Among these, *Saprospirae*, *Cytophagia*, *Betaproteobacteria* and *Synechococcophycideae* were dominant in Svalbard; *Sphingobacteria* in Northern Sweden; *Sphingobacteria* and *Saprospirae* in Greenland; *Saprospirae*, *Betaproteobacteria* and *Alphaproteobacteria* in Iceland (Fig. 2b and Supplementary Table 2).

In contrast, these biogeographic patterns were not observed for the snow algae. Our results demonstrate that the snow algal community composition and their relative abundance in all studied Arctic sites was highly similar (Fig. 2c,d), despite the large distances, physico-chemical characteristics and associated bacterial composition differences between sites. We show that the snow algae are cosmopolitan. This is in contrast to recent molecular studies, which suggest that in other terrestrial habitats, and even within a specific habitat, micro-eukaryotes show strong biogeography^20^,^25^,^26^,^27^. Our data reveal a very low algal diversity. Six taxa make up >99% of the algal communities (Fig. 2c,d and Supplementary Tables 3 and 4) and all have similar relative abundance values across all samples. The uncultured *Chlamydomonadaceae* (2) was the most abundant species (39–75%, Fig. 2d), followed by *Chloromonas polyptera* (10–26%), *Chloromonas nivalis* (3–13%), *Chloromonas alpina* (0–1%), the uncultured *Chlamydomonadaceae* (1) and *Raphidonema sempervirens* (1–18%). The small variance in the algal data between sites (Fig. 2c) was mainly caused by changes in relative abundance of the uncultured *Chlamydomonadaceae* (2), *Chloromonas polyptera* and *Raphidonema sempervirens*. However, none of the samples clustered according to locations, and no significant differences were found between locations for most of the algal species. The exceptions were *Chloromonas nivalis*, which showed a higher relative abundance in samples from Greenland in comparison to Svalbard, and the uncultured *Chlamydomonadaceae* (2), which had a higher relative abundance in Svalbard in comparison to Iceland (full details of the eukaryotic and archaeal community compositions and diversity indexes can be found in Supplementary Tables 5–8).

**Figure 3:**
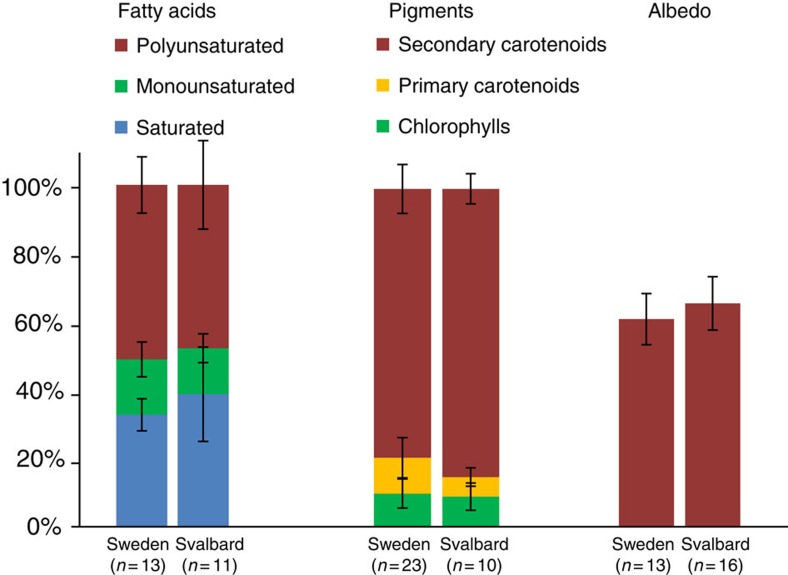
Algal fatty acid and pigment composition and albedo values. Comparison between average fatty acid and pigment compositions with average surface albedo (all in % of total) for all Svalbard and Northern Sweden sites. Error bars are standard deviations (for full details see Supplementary Tables 1, 3 and 10).

The homogeneous algal community composition described above was also mirrored in the similar composition of algal cell biomass, fatty acids and pigments with no significant differences between Svalbard and Northern Sweden (Fig. 3 and Supplementary Tables 9–11). On average, between 10^3^ and 10^4^ red pigmented algal cells per ml were present in our red snow samples. Despite the large variations in environmental parameters, no significant differences were found for cell numbers, cell sizes or total algal biomass (Supplementary Tables 3 and 9). Similarly, the fatty acid compositions in all analysed samples were similar with no statistically relevant differences between locations (Supplementary Tables 3 and 10). On average, ∼45–50% of all fatty acids were made up of polyunsaturated fatty acids, whereas saturated fatty acids comprised ∼30–40% and monounsaturated fatty acids were the least abundant (∼10–15%; Fig. 3 and Supplementary Table 10). The high content of polyunsaturated fatty acids likely demonstrates their role as cryo-protectants, helping algal cells to maintain membrane fluidity and preventing intracellular ice crystal formation^30^. The production of fatty acids is often linked to pigments^14^, which play the dominant role in changing the albedo. All samples were characterized by a high content of secondary carotenoids (∼70–90%), which are synthesized by the snow algae as a protection mechanism from high levels of irradiation, and with no significant differences between locations. The dominant secondary carotenoid was trans-astaxanthin (Supplementary Table 11). The remainder of the analysed pigments were typical primary carotenoids (up to 24%) or chlorophylls *a* and *b* (up to 55%; Supplementary Table 11).

### Local environment affects bacteria but not algae

Changes in physico-chemical conditions are known to control variations in microbial diversity in the environment^21^,^31^, yet for snow settings the importance or magnitude of these effects and whether they cause any biogeographical patterning are largely unknown. Our results show that the four chosen geographic locations differed substantially in the concentrations of essential nutrients, carbon species, trace elements (both dissolved and solid forms; Supplementary Tables 12 and 13) and mineralogy (Supplementary Table 14). Hence, they represent a good range of differing local snow environments across the Arctic. Dissolved organic carbon (DOC) concentrations varied significantly between locations (Supplementary Fig. 1 and Supplementary Tables 3 and 12), with up to five times higher values in snow from Northern Sweden in comparison to Svalbard. In contrast, concentrations of easily leachable elements (Ca, Cl, Mg, Mn, Na and K) were on average 10 times higher in red snow from Svalbard in comparison to Northern Sweden (Supplementary Table 12), whereas in Iceland the red snow samples contained up to 100 times higher iron concentrations than any of the other localities. These differences appear to be linked to the higher concentrations of more easily dissolvable mineral phases in Svalbard in comparison to Northern Sweden, and the higher Fe content in basaltic rocks from Iceland in comparison to the other sites (Supplementary Table 3). However, none of the essential nutrients (that is, NO_3_, PO_4_) showed any statistically relevant differences among locations (Supplementary Tables 3 and 12).

Mirroring the DOC trend, the total particulate carbon (TC) values as well as the total solid phase carbon to nitrogen (C/N) and carbon to phosphorous (C/P) ratios (Supplementary Table 13) were on average two or three times higher in Northern Sweden in comparison to Svalbard, whereas the δ^13^C values of bulk organic matter were significantly lower in Svalbard in comparison to Northern Sweden. In both locations, the C/N ratios were below the Redfield ratio but C/P values below Redfield were only present in samples from Northern Sweden (Supplementary Table 13). The high TC, DOC and δ^13^C values in the snow samples from Northern Sweden likely document a higher amount of allochthonous carbon potentially derived from higher plants, and the large amounts of pine pollen blown onto the glaciers and snow fields from the lower parts of the Tarfala valley. However, all red snow samples, regardless of location, were characterized by similar, predominantly negative organic δ^15^N values indicative of an atmospheric source (Supplementary Table 13).

Geochemical and mineralogical parameters varied dramatically between locations, yet no correlations between algal species distribution and these characteristics were found (Supplementary Fig. 2). This suggests that the uniform algal species composition remained unaffected by and independent of the local geochemical and mineralogical parameters in each site.

In contrast, our data show a clear links between the bacterial community composition and geochemical parameters, with the most positive correlation found between the carbon species (TC and DOC; Supplementary Fig. 3) and *Sphingobacteria*. This is not surprising as *Sphingobacteria* are known to be capable of degrading complex organic structures^32^, and their abundance in Northern Sweden is consistent with the local high DOC and TC values (Supplementary Tables 12 and 13). In all samples and in total contrast to the algae, the bacteria are seemingly subjected to a much higher location-specific selection pressure and appeared more affected by the availability of allochthonous carbon and the local geology.

### Algae decrease surface albedo

**Figure 4:**
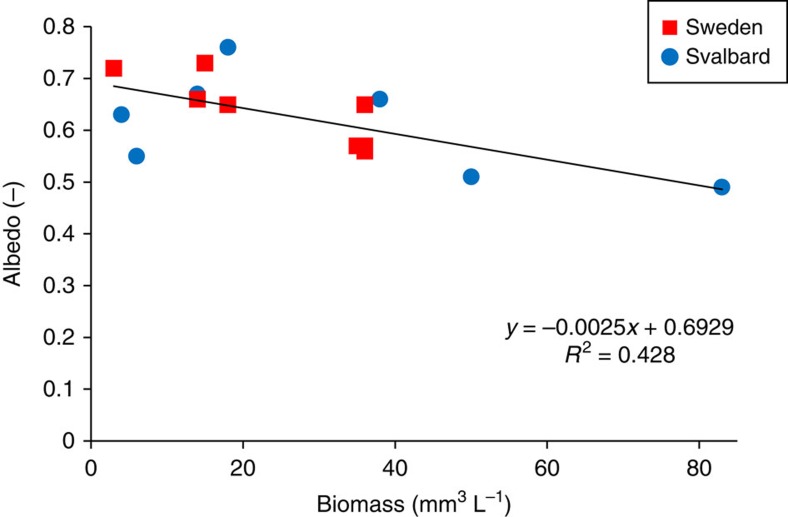
Algal biomass and albedo. Plot shows a significant negative correlation (Pearson correlation factor: *r*=−0.65, *P*=0.008) between algal biomass and surface albedo measured in red snow sites in Svalbard and Northern Sweden. This underpins the role of red pigmented snow algae in decreasing surface albedo and in turn melting.

The above documented high algal biomass primarily made up of highly red pigmented algae, will invariably affect the amount of light that is reflected from the surface of snow fields. Our albedo measurements (Table 1 and Supplementary Table 1) showed a clear decrease in surface albedo in comparison to algal-free snow sites (0.90±0.05; Table 1)^11^,^33^. The measured decrease where red pigmented algae were present was similar in all sites, independent of the local environment with albedo values reaching between ∼0.50 and 0.75 (Supplementary Table 1). In addition, we found a significant (*P*=0.008) negative correlation between algal biomass and surface albedo (Fig. 4), which clearly supports our assertion of the crucial role of red pigmented snow algae in decreasing surface albedo and increasing melting. This is also on par with the results by Painter *et al*.^34^ and Aoki *et al*.^35^, who showed that the strong light absorption is due to algal pigments in the 400–600 nm (carotenoids and chlorophylls) and 600–700 nm (chlorophylls) range, which is much stronger in comparison to absorption by mineral dust or black carbon if biomass is as high as in our documented red algal blooms.

The above described ubiquitous distribution, low diversity and similarity in snow algal community compositions and metabolic functions combined with the analogous values measured for the red snow algae induced albedo reduction (Supplementary Table 1), allow us to compare the impact that the red pigmented snow algae have on albedo in comparison to snow surfaces free of algae over an estimated 100-day scenario. At the beginning of a melting season, we assume all glacier surfaces are covered by dry clean snow. Using values for albedo for such snow from the literature^2^,^22^,^36^,^37^ (0.90±0.05; Table 1) allowed us to linearly integrate the change in albedo of snow colonized by red pigmented algae versus algae-free snow surfaces for the season-long transition from dry clean snow to wet clean snow (0.75±0.05) and to red snow (0.65±0.12; Supplementary Fig. 4). We show a square root dependence between albedo values and time and applied a simple one-dimensional moving boundary approach to our data^38^.

Our fit of the integrated albedo change with time (100 days) shows a 13% larger effect in the presence of red pigmented algal blooms in comparison to clean snow that has undergone a purely physical albedo change due to melting, and change of snow crystal size and structure. This 13% integrated change in albedo is an estimate for the overall effect of snow algal communities during an entire melt season and compares well with the single time-point albedo reduction of up to 20%, that we and others have previously measured on red pigmented algal snow sites^11^,^39^. Moreover, with further melting dirty ice and cryoconite holes will be exposed earlier and their albedo values can drop by an additional ∼20% to 0.34±0.15. This will likely culminate in even higher melt rates, which has also recently been shown in laboratory experiments^40^.

A quantitative value for the area of Arctic glaciers and the Greenland Ice Sheet covered by snow algae during a melt season is still lacking. However, as we infer from our data, melting is one major driver for snow algal growth. Extreme melt events like that in 2012, when 97% of the entire Greenland Ice Sheet was affected by surface melting^41^, are likely to re-occur with increasing frequency in the near future as a consequence of global warming^42^. Moreover, such extreme melting events are likely to even further intensify the effect of snow algae on surface albedo, and in turn melting rates^43^.

With this work, we show that snow algae are ubiquitous and have little diversity across the Arctic, despite variations in environmental parameters that significantly impact the bacterial community composition. Although we may not have captured all environmental parameters, the patterns we observed occur on all studied glaciers regardless their geochemical and mineralogical compositions. Further investigations are needed to explore the validity of these findings for mid latitudes and the southern hemisphere. However, red snow is a ubiquitous phenomenon in Arctic sites (glaciers and permanent snow fields). The similarity in snow algal community composition, metabolic function and impact on albedo of snow habitats allows the upscaling of our findings and make predictions about the influence of snow algae on melt rates of glaciers across the Arctic. Our data show that the overall decrease in snow albedo by red pigmented snow algal blooms over an entire melt season can be 13%, likely leading to earlier exposure of dirty ice with an even lower surface albedo culminating in a further increase in melt rates. Our work paves the way for a universal model of algal–albedo interaction and a quantification of additional melting caused by algal blooms to be included in future climate models.

## Methods

### Field sites

A total of 40 red snow samples was collected from four well-separated Arctic locations on 16 glaciers and permanent snow fields: Svalbard (SVA, *n*=12), Northern Sweden (TAR, *n*=24), Greenland (MIT, *n*=2) and Iceland (ICE, *n*=2; Fig. 1; Supplementary Table 1). These localities were chosen as they represent different geographical settings including low (67.9°N) versus high (78.9 N) latitude, low (150–400 m) versus high (∼1,200–1,400 m) elevation, and maritime versus continental settings. Vestre Brøggerbreen, Midtre Lovénbreen, Austre Brøggerbreen, Pedersenbreen, Austre Lovénbreen and Feiringbreen in Svalbard were sampled in July and August 2013. Samples from Storglaciären, Rabot, Liljetopsrännan, SE-Kasskasatjåkkå, Björling and nearby permanent snow fields in Northern Sweden were collected in July 2013 and July 2014. Mittivakkat glacier in Greenland and the glacier Drangajökull and permanent snow field Laugafell in Iceland were sampled in July 2012. Red snow samples were collected late in the melt season as those are the typical snow algal blooms that will have the largest effect on albedo.

### Field sampling and measurements

All sampling, field measurements and most analyses have previously been described in full detail^11^,^33^. Here we summarize previously employed methods and give full details of new methods. At each sampling site, we measured pH, conductivity and temperature with a daily calibrated metre (Hanna instruments, HI 98129) before sampling. Photosynthetic active radiation, ultraviolet radiation and surface albedo (400–700 nm range) were measured using a radiometer (SolarLight, PMA2100) with specific photosynthetic active radiation (PMA2132), ultraviolet-A (PMA2110) and ultraviolet-B (PMA2106) sensors. Albedo was calculated by taking the ratio of reflected to incident radiation (400–700 nm range) and measuring the values always in the same position to the sun. The reading of the sensor was not affected by shading by the observer. Measurements were carried out with the sensors held at 30 cm above the snow surface (field of view 160°). At first, the sensor was pointed upwards (incident radiation) and then towards the snow surface (reflected radiation). Five measurements for incident and reflected radiation were acquired each, and the average was taken to avoid measuring bias. The standard deviations for each measurement set was below 10%. Data of the relative contribution of pigments, other light impurities (that is, mineral dust, black carbon) and snow metamorphism is lacking^42^. However, based on qualitative microscopic observations in the field, particularly mineral impurities (most often light coloured quartz and feldspars) were less important in changing albedo measurements in red snow surface samples in comparison to the pigment distribution. Moreover, Aoki *et al*.^35^ and Painter *et al*.^34^ showed that red snow has much higher light absorption below 600 nm because of the algal pigments in comparison to mineral dust or black carbon. Samples were collected in sterile centrifuge tubes or sterile *Whirl-Pak* bags and in pre-ashed glass jars (450 °C, >4 h) for organic analyses. All samples were slowly melted at room temperature within ∼6 h, and processed and preserved (for example, filtered, acidified) within ∼8 h after collection. Samples for DNA and organic analyses were flash-frozen in liquid nitrogen and stored at −80 °C until analysed. Inorganic samples were stored cold (4 °C) and in the dark. All analyses were carried out in the Cohen Laboratories at the University of Leeds unless stated otherwise.

### Aqueous analyses

Aqueous analyses were carried out by Ion Chromatography (Dionex, anions), by inductively coupled plasma mass spectrometry (Agilent, cations, at the University of Sheffield), on a total organic carbon analyser (Shimadzu, for DOC contents, DOC, at the Plymouth University), and by segmented flow-injection analyses (AutoAnalyser3, Seal Analytical, dissolved phosphate).

### Particulate analyses

Particulates in the samples were analysed for δ^15^N and δ^13^C by a Vario Pyro Cube elemental analyser (Elementar Inc) coupled to an Isoprime mass spectrometer. Samples were combusted in tin capsules at 1,150 °C, and gases were separated using temperature-controlled adsorption/desorption columns. Carbon analyses were calibrated with in-house C4-sucrose and urea standards assigned values of −11.93‰ and −46.83‰, respectively via calibration with the international standards LSVEC (−46.479‰), CH7 (−31.83‰), CH6 (−10.45‰) and CO-1 (+2.48‰). Nitrogen isotope values were calibrated using the international standards USGS-25 and USGS-26 with assigned values of −30.4‰ and +53.7‰, respectively. Total carbon, total nitrogen and total sulphur were derived from the thermal conductivity detector in the elemental analyser and calibrated using a sulphanilamide standard. Particulate phosphorus was extracted by ashing of the samples at 550 °C for 2 h and incubating in 1 M HCl for 16 h according to extraction step V in Ruttenberg *et al*.^44^.

### Algal biomass

Algal cells were imaged on a Leica DM750 microscope equipped with a × 63objective and counted with a haemocytometer in triplicate. For cell size analyses, 100 cell diameters per sample were measured in ImageJ. Cell volumes were calculated assuming a perfect spherical shape (V=4/3*π*r^3^). Total algal biomass was calculated using the average cell volume and cell abundance.

### Pigment analysis

Carotenoid and chlorophyll contents in the samples were quantified by high-pressure liquid chromatography (HPLC) and a modified carotenoid/chlorophyll-specific extraction protocol^45^. Cells were disrupted by shock freezing in liquid nitrogen for 10 min followed by grinding with a Teflon mortar and pestle. The resulting powder was re-suspended in 1 ml of dimethylformamide and 1.0 mm glass beads and horizontally shaken on a laboratory shaker (MoBio Vortex Genie 2) at maximum speed (3,000 r.p.m.) for 10 min, followed by centrifugation for 5 min at 10,000 r.p.m. The supernatant was separated from the debris by filtering through a 0.45-μm Teflon filter and the filtrate was mixed with methanol (25 vol%).

Extracted samples were analysed immediately on an Agilent Technologies 1200 Infinity HPLC instrument with a gradient pump, an autosampler, a variable wavelength detector and ODS Hypersil column (250 × 4.6 mm^2^; 5 μm particle size). Two solvents were used: solvent A consisted of a mixture of acetonitrile/water/methanol/hexane/tris buffer at ratios of 80:7:3:1:1, whereas solvent B was a mix of methanol and hexane at a ratio of 5:1. The HPLC was run at a flow rate of 1 ml min^−1^, with an injection volume of 25 μl. Spectra were recorded from 200 to 800 nm. Chromatograms were quantified at 450 nm for carotenoids and 660 nm for chlorophyll *a* and *b*. Run time was 60 min. The protocol required a 15-min run with 100% of solvent A followed by a linear gradient from 100% solvent A, to 100% solvent B between 32 and 45 min, and finally with 15 min of column re-equilibration through a 5-min linear gradient from solvent B back to 100% solvent to A, followed by a further column conditioning with 100% solvent A for 10 min. The following commercially available standards were used for peak identification: chlorophyll *a*, chlorophyll *b* (Sigma), violaxanthin, neoxanthin, antheraxanthin, lutein, β-carotene, *trans*-astaxanthin and *cis*-astaxanthin (Carotenature).

### Fatty acids analysis

Fatty acids were extracted according to the method described by Wacker and Martin-Creuzberg^46^. Briefly, 20 ng of internal standard (tricosanoic acid methyl ester) were added to each sample, followed by ultrasonic extraction using dichloromethane:methanol (2:1 (v:v)), and centrifugation to remove particulates and evaporation of solvent from the supernatant. Fatty acids were transesterified by adding methanolic HCl to the dried extract and heating at 60 ° C for 20 min. After cooling, fatty acid methyl esters were extracted in isohexane, the solvent was removed under nitrogen and the sample resuspended in isohexane for analysis.

Analysis of fatty acid methyl esters was carried out using a Trace 1300 gas chromatograph with flame ionization detector (Thermo Scientific), equipped with a non-polar-fused silica capillary column (CPSil-5CB, 50 m × 0.32 mm × 0.12 mm, Agilent Technologies). Samples (1 μl) were injected in splitless mode, with the injector maintained at 200 °C. Carrier gas was helium, with a constant flow rate of 1.5 ml min^−1^. The following temperature programme was used: initial temperature 40 °C, rising to 140 °C at 20 °C min^−1^, then rising to 240 °C at 4 °C min^−1^, holding at 240 °C for 5 min. Fatty acid methyl esters were identified by comparison of retention time with those of reference compounds (Supelco) and by gas chromatography mass spectrometry (GC–MS). GC–MS was carried out using the gas chromatograph and column previously described, with identical operating conditions, coupled to an ISQ mass spectrometer (Thermo Scientific). The transfer line and the ion source were maintained at 300 °C. The emission current was set to 50 mA and the electron energy to 70 eV. The analyser was set to scan at *m*/*z* 50–650 with a scan cycle time of 0.6 s.

### DNA sequencing

Total DNA was extracted from pelleted biomass using the PowerSoil DNA Isolation kit (MoBio Laboratories). 16S rRNA genes were amplified using bacterial primers 27F (5′-AGAGTTTGATCMTGGCTCAG-3′) and 357R (5′-CTGCTGCCTYCCGTA-3′; tagged with the Ion Torrent adapter sequences and MID barcode) spanning the V1-V2 hypervariable regions. 18S rRNA genes were amplified using the eukaryotic primers 528F (5′-GCGGTAATTCCAGCTCCAA-3′) and 706R (5′-AATCCRAGAATTTCACCTCT-3′; Cheung *et al*., 2010 (ref. 48); tagged with the Ion Torrent adapter sequences and MID barcode) spanning the V4-V5 hypervariable region. PCRs were performed using Platinum PCR SuperMix High Fidelity according to the manufacturer's protocols. Initial denaturation at 95 °C for 5 min was followed by 30 cycles of denaturation at 95 °C for 30 s, annealing at 60 °C for 30 s and elongation at 72 °C for 30 s. Final elongation was at 72 °C for 7 min. Archaeal 16S rRNA genes were amplified following a nested PCR approach. The first PCR reaction was carried out using primers 20F (5′-TCCGGTTGATCCYGCCRG-3′) and 915R (5′-GTGCTCCCCCGCCAATTCCT-3′). Initial denaturation at 95 °C for 5 min was followed by 35 cycles of denaturation at 95 °C for 30 s, annealing at 62 °C for 30 s and elongation at 72 °C for 180 s. Final elongation was at 72 °C for 10 min. The PCR product was used as template for the second PCR reaction with primers 21F (5′-TCCGGTTGATCCYGCCGG-3′) and 519R (5′-GWATTACCGCGGCKGCTG-3′; tagged with the Ion Torrent adapter sequences and MID barcode) spanning the V1-V2 hypervariable region. Initial denaturation at 95 °C for 5 min was followed by 30 cycles of denaturation at 95 °C for 30 s, annealing at 60 °C for 30 s and elongation at 72 °C for 30 s. Final elongation was at 72 °C for 7 min. Detailed information on the sequencing primers can be found in the Supplementary Information. All PCRs were carried out in triplicates to reduce amplification bias and in reaction volumes of 1 × 25 μl and 2 × 12.5 μl. All pre-amplification steps were done in a laminar flow hood with DNA-free certified plasticware and filter tips. The pooled amplicons were purified with AMPure XP beads (Agencourt) with a bead–to-DNA ratio of 0.6 to remove nucleotides, salts and primers. Quality, size and concentration were determined on the Agilent 2100 Bioanalyser (Agilent Technologies) with the High-Sensitivity DNA kit (Agilent Technologies). Sequencing was performed on an Ion Torrent Personal Genome Machine using the Ion Xpress Template Kit and the Ion 314 or Ion 316 chips following the manufacturer's protocols. All PCR amplifications and sequencing were carried out at the Aberystwyth University and the University of Bristol. The raw sequence data were processed in QIIME^47^. Barcodes and adapter sequences were removed from each sequence. Filtering of sequences was performed using an average cutoff of Q20 over the full sequence length (350 bp). Reads shorter than 200 bp were removed. Operational taxonomic units (OTUs) were picked *de novo* using a threshold of 97% identity. Taxonomic identities were assigned for representative sequences of each OTU using the reference databases Greengenes for bacteria and archaea. The Silva database (ref. 49; extended with additional 223 sequences of cryophilic algae kindly provided by Dr Thomas Leya from the CCCryo—Culture Collection of Cryophilic Algae, Fraunhofer IZI-BB) was used for eukaryotes. Data were aligned using PyNAST and a 0.80 confidence threshold. Singletons were excluded from the analysis. For bacterial sequence matching, plant plastids were removed from the data set before further analysis. For eukaryotic sequence matching, *Chloroplastida* were pulled out of the data set and stored in a separate OTU table. In order to focus upon algal diversity, sequences matching *Embryophyta* (for example, moss, fern) were removed from the data set. For archaea, sequences matching bacteria were removed. Finally, for further analyses, samples were rarefied to the minimum library size and Shannon indices were calculated in QIIME. All analyses were conducted at the 97% OTU level. A matrix of each OTU table representing relative abundance (raw data) was imported into PAST v3.06 (ref. 50) for multivariate statistical analyses (principal component analysis, canonical correspondence analysis) and Pearson correlations. One-way analysis of variance test was done in SPSS v19 (IBM).

### Sequencing primers

Primers targeting the 18S rRNA gene were chosen because there are more sequences in the databases for green algae (that is, *Chlorophyta*, *Charophyta*) than for rbcL or internal transcribed spacer (ITS). Before sequencing, we carried out an *in-silico* investigation including 18S rNRA sequences from 218 snow and permafrost algae in order to make sure that the chosen primers are suitable for green algae and that there is enough variability in the chosen region (v4-v5) to distinguish between species.

Previous studies^51^ have found that one primer pair is not sufficient to recover all eukaryotic groups in one sample. However, we chose our primer pair based on one group we were specifically targeting, that is, the green algae. We do not claim to have equally recovered all other groups among the micro-eukaryotes such as fungi or the ‘SAR'-group. Furthermore, they found that libraries derived from different primer pairs grouped together for individual samples with no significant differences. Based on our *in silico* test of 218 snow and permafrost algae and the rarefaction curves (Supplementary Fig. 6), we are fairly confident that the choice of our primer pair has resulted in a good coverage of the algal diversity. However, we acknowledge that PCR-based approaches will always introduce a certain amount of bias.

This is similar for the archaea, which show no biogeographical patterns in our samples. The primers used are specific for archaea and since they are not the focus of this study and only the associated microbiome, we did not explore other primer possibilities. However, the results match what other studies have found before in cryo-environments^28^,^52^.

### Overall sampling design

All samples for DNA and aqueous analyses were analysed in exactly the same way for all samples from Greenland, Iceland, Svalbard and Sweden. Pigment and fatty acid data are only shown for the samples from Svalbard and Sweden because for Greenland and Iceland these data have previously been published^11^,^33^. The samples from Greenland have been excluded from the comparison here because the pigment and fatty acid data have been collected and quantified in a different way. The pigments were normalized to chlorophyll *a*, whereas in all other study area they were quantified with the appropriate pigment standards. The fatty acid components were analysed by GC–MS, whereas all samples from Svalbard and Sweden were also quantified by flame ionization detection. The pigment and fatty acid data from Iceland were also excluded from the comparison, as in all samples large amounts of moss (identified by microscopy and DNA sequences) that could not be separated from the algae before pigment and fatty acid extraction were present. This moss contribution would strongly ‘skew' the data and thus these were excluded.

In addition, only selected samples in Greenland^11^ and Iceland^33^ were included in the comparison. This is because at both sites samples were collected at different stages in the melt season. The study in Greenland was conducted at the onset of melting and over a 3-week period when snow algae just started to bloom and a decrease in relative chlorophyll content and increase in carotenoid content could be observed. This led to our conclusion that there is a great heterogeneity in pigment composition both in space and time^11^. However, the few samples collected at the end of the study showed similar carotenoid contents. This end-of-season homogeneity in the red snow samples was the reason why in the current study we focused solely on samples from late in the melt season, which is the dominant red snow stage with the largest impact on albedo. Thus, we only included two DNA samples from Greenland. Similarly in Iceland^33^, most of the samples were collected earlier in the year (June—July) and those samples were described as less ‘typical' of red snow patches^33^. Off all samples from Iceland again only the two samples that were collected late in the melt season and therefore matched the conditions of the samples in the current study were used for comparisons.

### Integrated albedo change

Using our mean, minimum and maximum measured albedo values for wet clean snow and red snow and literature data^22^ for clean dry snow, we used a simple one-dimensional moving boundary approach that allows us to predict the effect of red pigmented snow algae on albedo. This approach is valid under the assumption that the snow and ice surfaces melt downwards relative to a fixed depth, and that at the same time such a change is accompanied by changes in albedo^38^. The parameters, equations and boundary conditions used are as follows:

Table 1 shows measured minimum, maximum and average albedo values for dry clean snow before the onset of melting^11^, wet clean snow (no visual presence of algae) at the onset of melting and red snow (full red pigmented snow algal bloom). We used these values to derive linear regressions for albedo changes over a 100-day melt season (Supplementary Fig. 4). A conservative 100-day scenario was chosen, as this encompasses all our albedo measurements in the current and previous studies (June—August)^3^,^11^,^33^. In addition, this also corresponds to the number of days with mean air temperatures above 0 °C in the same period (Ny Alesund: 116 days in 2013 and 105 days in 2014, kindly provided by Dr Marion Maturilli and Siegrid Debatin, AWI; Storglaciären: 132 days in 2013 and 108 days in 2014; kindly provided by Dr Peter Jansson, Stockholm University; data are also publicly available at http://bolin.su.se/data/tarfala/). We compare a benchmark case of purely physical-driven albedo change (that is, changes in snow crystal sizes and shapes and increasing water content, scenario 1) with albedo change due to red pigmented algal growth (scenario 2).

Scenario 1 considers the transition over 25 days from clean snow to a wet melting surface without algal growth and with an albedo of 0.80 (a minimum value), which with continued melting results in an albedo of 0.75 (an average value) after 50 days and 0.70 (a maximum value) after 100 days (Table 1). Our benchmark case (scenario 1) shows albedo (*α*) changes with time and fits the equation:




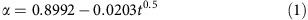




Scenario 2 considers the transition from clean snow to a surface where the growth of algae after 25 days produces light red snow with an albedo of 0.77 (a minimum value), and continued melting produces darker red snow with an albedo of 0.65 (an average value) after 50 days and 0.53 (a maximum value) after 100 days (Table 1). The albedo changes with time for this scenario fit the equation:




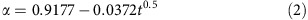




These two equations can be integrated to obtain the cumulative effects of albedo (*α*) changes with time to give:




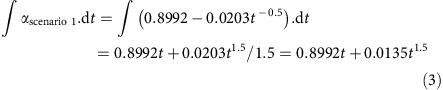







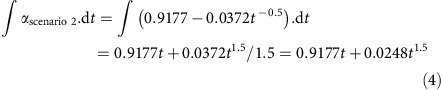




Subtracting equations (3) and (4) gives




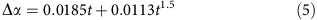




which represents the albedo changes attributable to algae growth alone. For a melt season of 100 days Δα=1.85+11.3=13.15≈13.

In order to assess the error of our analysis, we carried out a sensitivity analysis using the data below (see also Supplementary Fig. 5 for details):




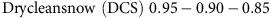







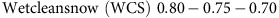







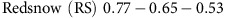




Comparing minimum and average albedo values:




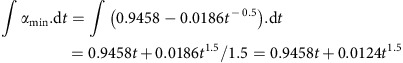







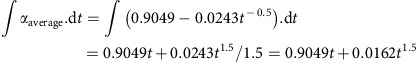




Subtracting gives Δ*α*=0.0409*t*–0.0038*t*^1.5^

So when *t*=100, Δ*α*=4.09–0.0038 × 1,000=4.09–3.8=0.29

Comparing average and maximum values:




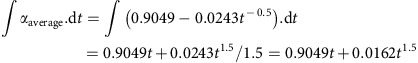







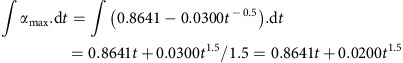




Subtracting gives Δ*α*=0.0408*t*–0.0038*t*^1.5^

So when *t*=100, Δ*α*=4.08–0.0038 × 1,000=4.08–3.8=0.28

So our sensitivity test is giving a crude range of ∼0.3 about the mean.

### Data availability

DNA sequences have been deposited to the European Nucleotide Archive (ENA) under accession number PRJEB10548. All other data are available in the Supplementary Information.

## Additional information

**How to cite this article:** Lutz, S. *et al*. The biogeography of red snow microbiomes and their role in melting arctic glaciers. *Nat. Commun.* 7:11968 doi: 10.1038/ncomms11968 (2016).

## Supplementary Material

Supplementary Information Supplementary Figures 1-6, Supplementary Tables 1-14 and Supplementary References.

## Figures and Tables

**Table 1:** Integrated albedo change.

	Average	Minimum	Maximum	Reference
Dry (winter) clean snow	0.90	0.95	0.85	37
Wet clean snow	0.75	0.80	0.70	11
Red snow	0.65	0.77	0.53	11, Supplementary Table 1

Average, minimum and maximum albedo values (in terms of decrease in albedo) for dry clean snow, wet clean snow and red snow used to derive the integrated albedo change over 100 days (Supplementary Fig. 4).
