# Supplementary material for: The biogeography of red snow microbiomes and their role in melting arctic glaciers
Source: Nat Commun. 2016 Jun 22;7:11968. doi: 10.1038/ncomms11968 (PMC4917964; doi:10.1038/ncomms11968)
Supplement: Supplementary Information — Supplementary Figures 1-6, Supplementary Tables 1-14 and Supplementary References. [file ncomms11968-s1.pdf]

Supplementary Figure 1: PCA of aqueous geochemical parameters

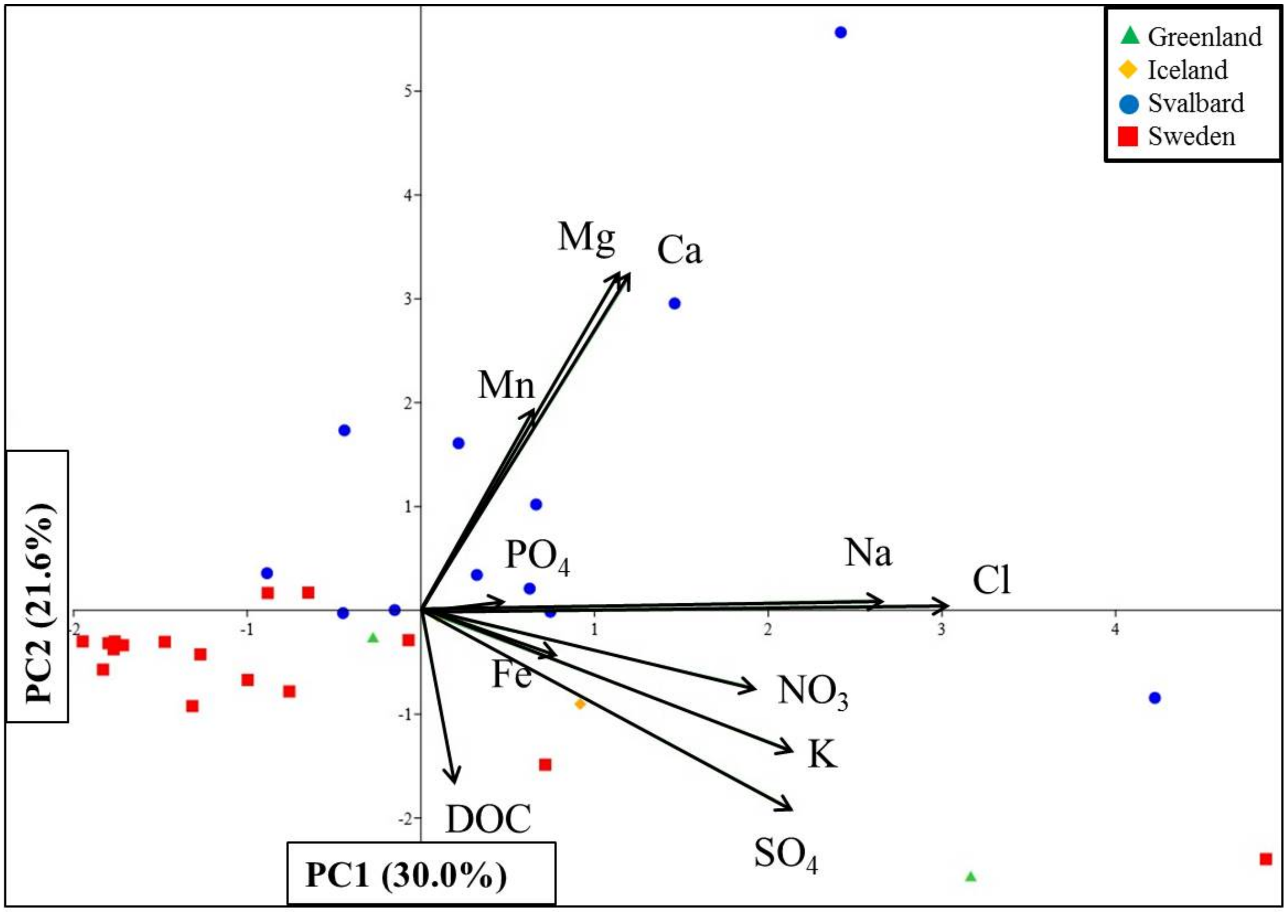

PCA of aqueous geochemical parameters revealing differences between locations. Samples cluster according to locations with Arctic Sweden showing a trend in higher DOC concentrations whereas higher Ca, Cl, Mg, Mn, and Na concentrations are matching the Svalbard samples.

Supplementary Figure 2: CCA for algal species and geochemistry

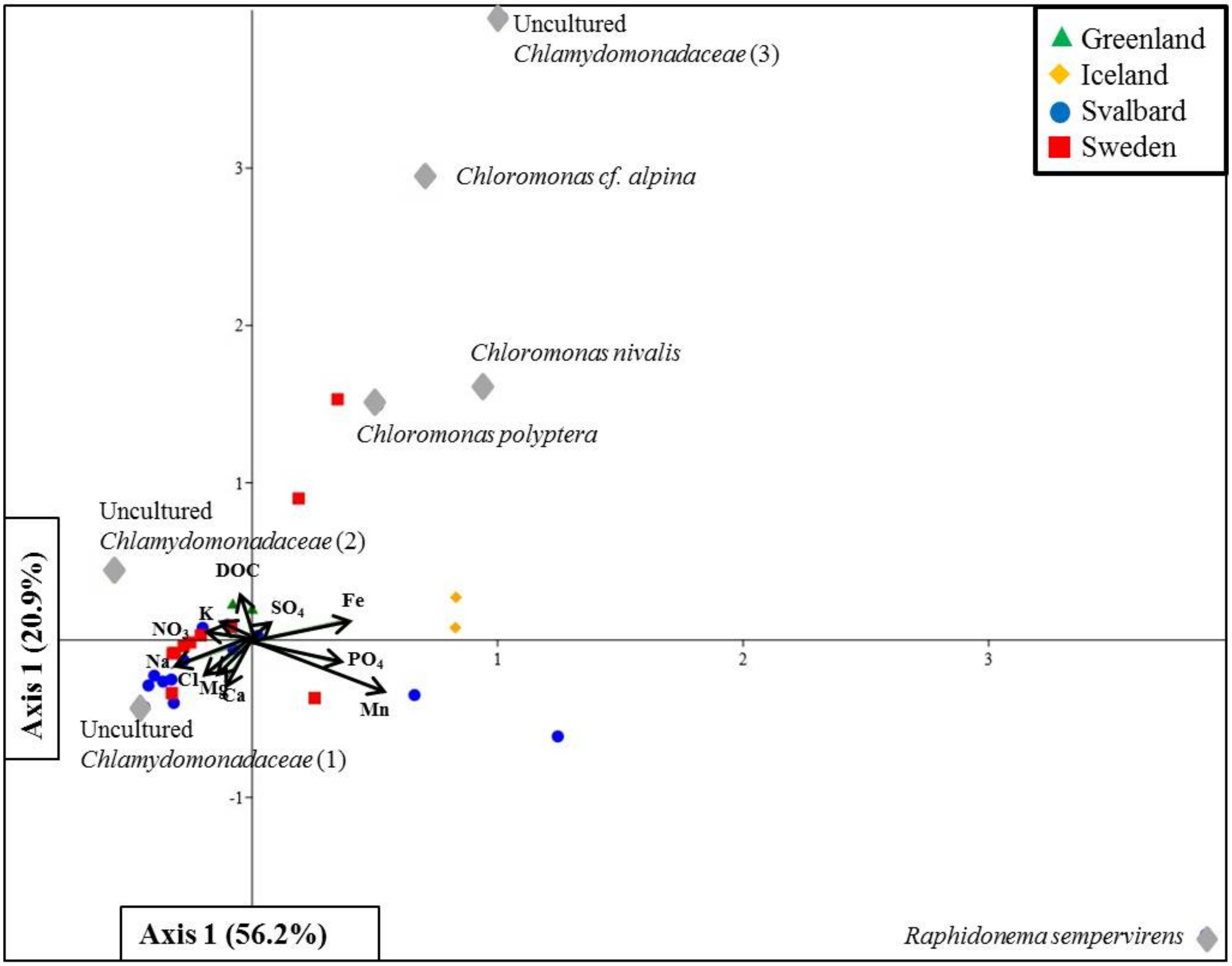

CCA for algal species (grey diamonds) and geochemistry (arrows) showing no clustering of samples and no trends for any of the samples or species with the analysed aqueous geochemical parameters.

Supplementary Figure 3: CCA for bacterial classes and geochemistry

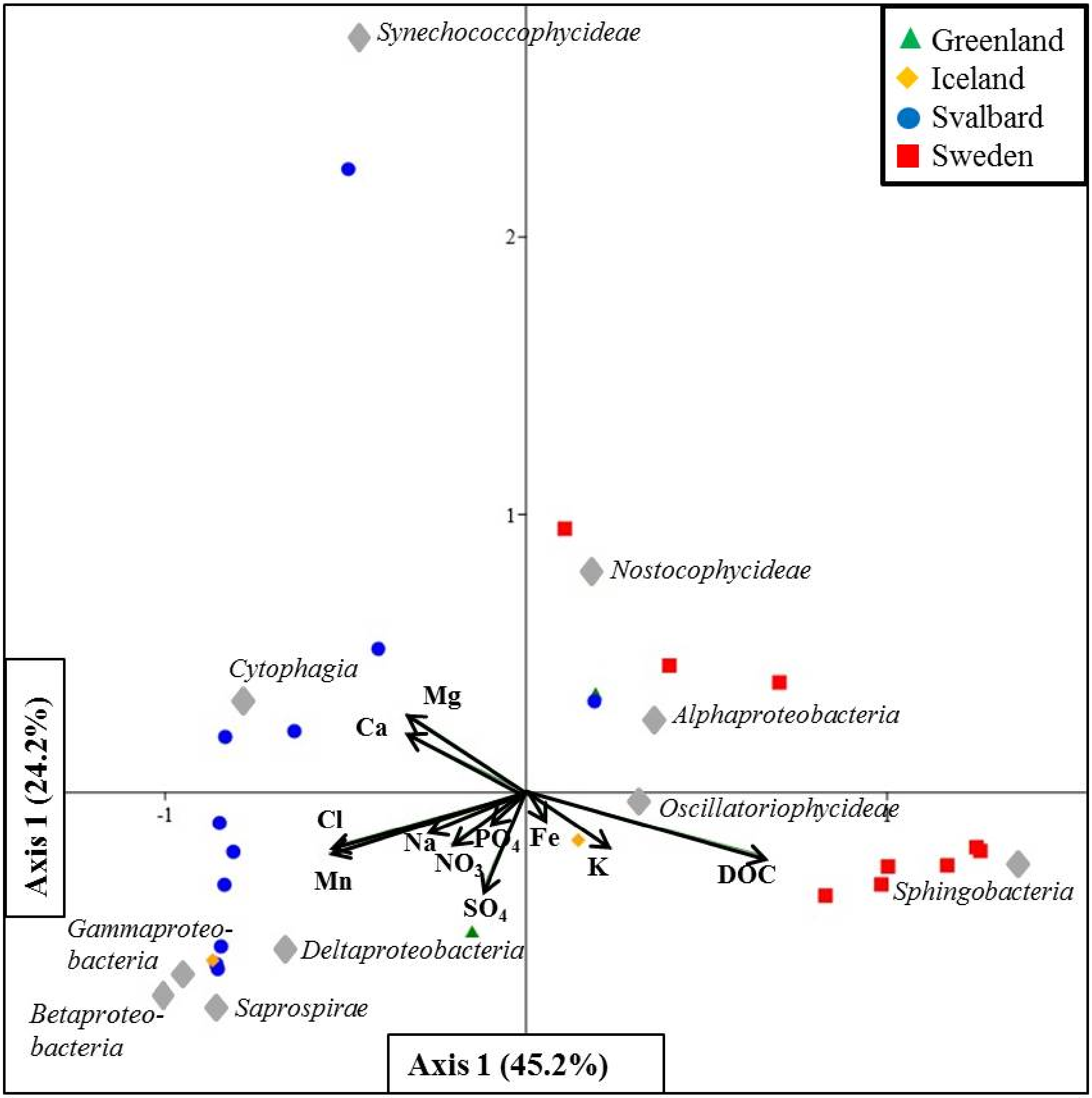

CCA for bacterial classes (grey diamonds) and geochemistry (arrows) showing a clustering of samples according to locations and a positive correlation between *Sphingobacteria* and DOC.

Supplementary Figure 4: Integrated albedo change

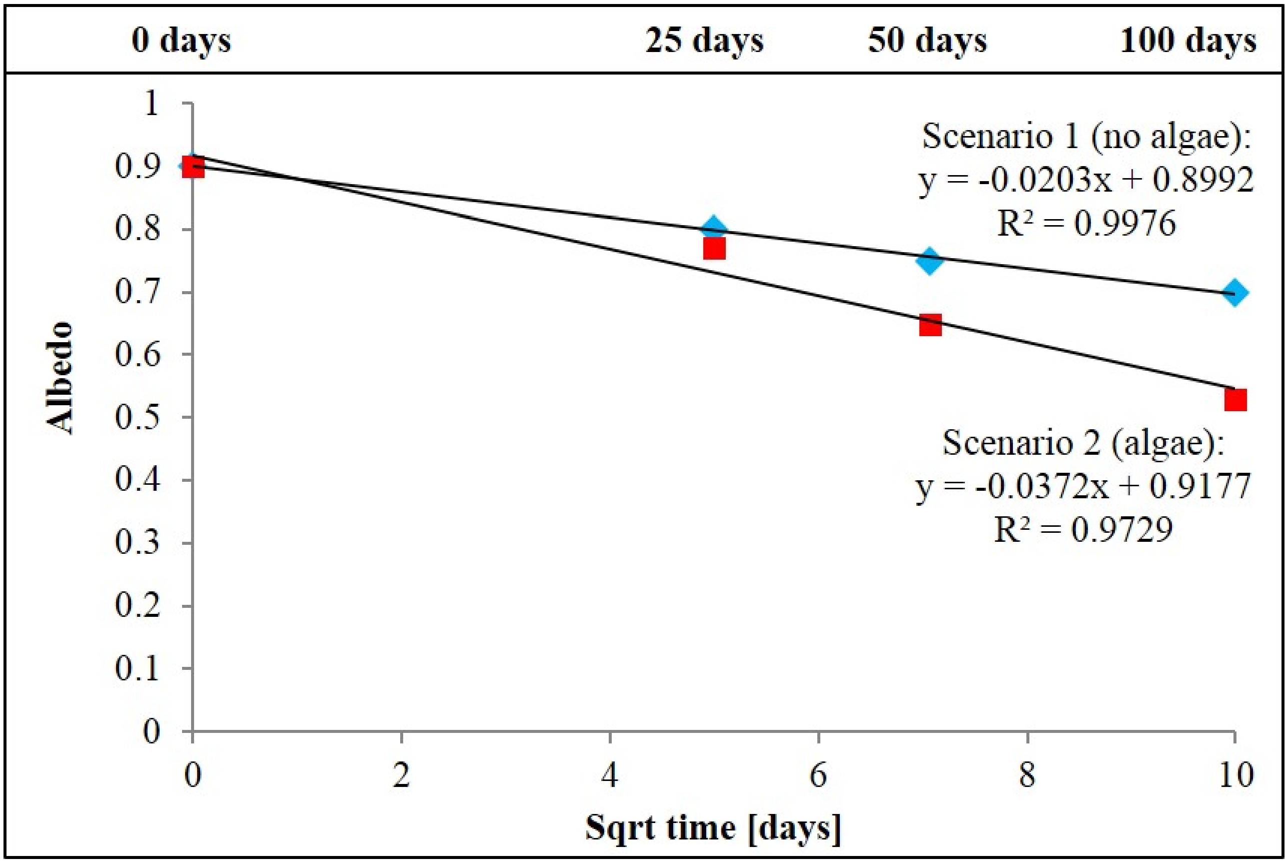

Output from the used one-dimensional moving boundary approach (based on our and literature data; see Table 1 in the main document) with the derived equations for the changes in albedo over 100 days of melting due to purely physical changes (blue diamonds) and due to the blooming of red pigmented algae (red squares). Scenario 1 (blue diamonds) represents a purely physical reduction in albedo values (α) due to melt induced changes in snow crystals shape and size, with no input from red pigmented snow algae (dry clean snow → wet clean snow). Scenario 2 (red squares) represents the changes in albedo over the same period, but due to the reduction in albedo caused by the presence of red pigmented snow algae (dry clean snow → red snow). For the origin of the used albedo data as well as the values used in our scenarios see Supplementary Table 1 and Table 1.

Supplementary Figure 5: Sensitivity analysis for integration albedo change fits

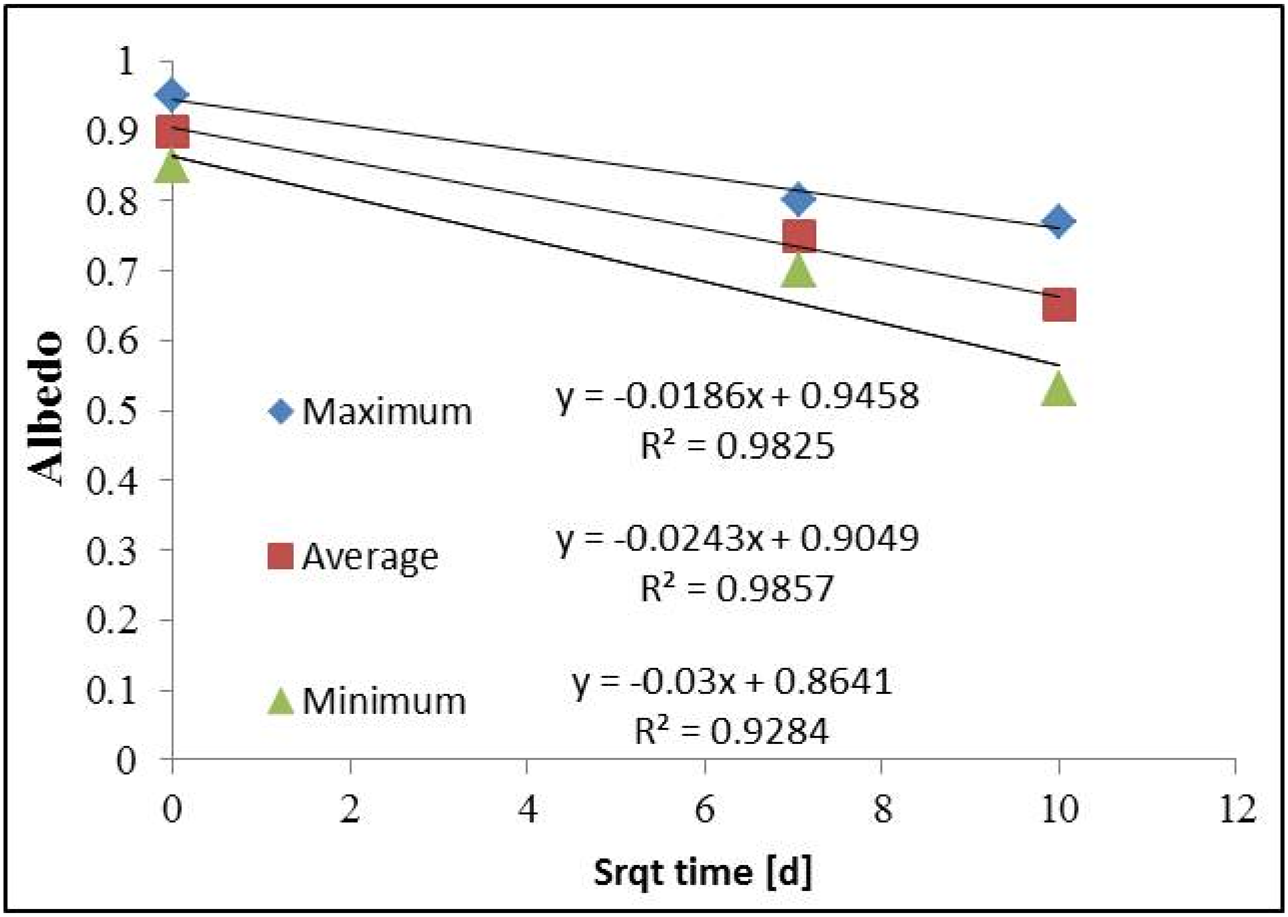

Sensitivity analysis for integrated albedo change fits as based on our and literature data (see Table 1 in the main document and Supplementary Table 1) with equations for maximum, average and minimum values for dry clean snow, wet clean snow and red snow (Table 1). The full sensitivity analysis is provided in the methods section at the end of the manuscript.

Supplementary Figure 6: Rarefaction curves for algal sequences

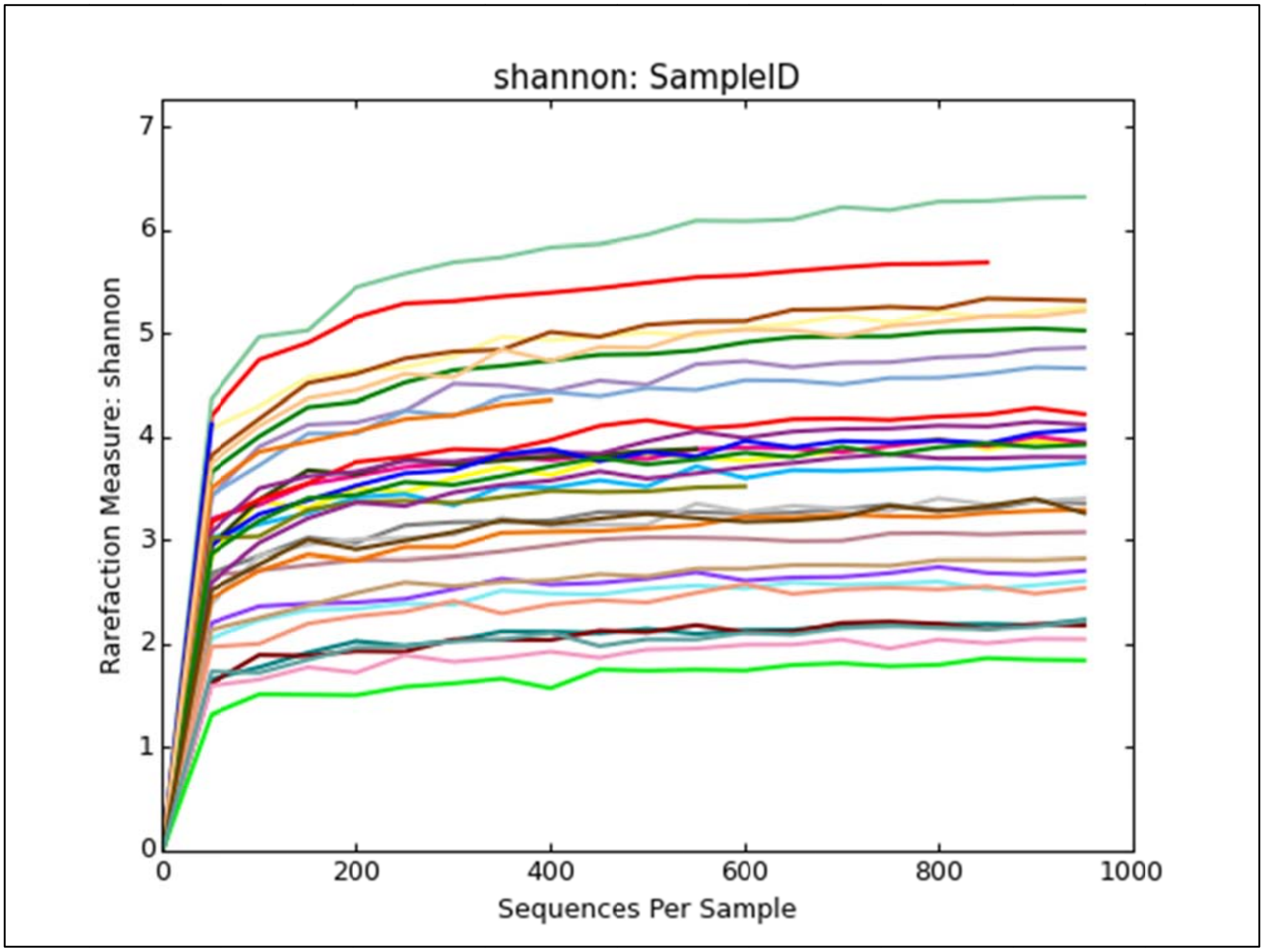

Rarefaction curves for all samples (algal sequences) suggesting a good coverage of the algal diversity. Samples were rarefied to the smallest library size and alpha diversity was estimated using Shannon indices.

Supplementary Table 1: Sample overview

Overview of samples, locations, coordinates and field measurements for red snow algae samples collected from Svalbard (SVA), Northern Sweden (TAR), Greenland (MIT) and Iceland (ICE). Only a few samples from Greenland and Iceland have been included in this study for the pan-Arctic comparison and many more data from other snow and ice habitat samples can be found in previous publications[1,2].

Sample ID	Location	Collection date	GPS location [UTM]	Elev. [m a.s.l.]	pH	Snow temp. [°C]	PAR [W/m²]	UV-A [W/m²]	UV-B [W/m²]	Albedo
**Svalbard, Norway**										
**SVA-13_2**	Vestre Brøggerbreen	20/07/2013	33H 0433169 E, 8759838 N	265	7.03	0	48			0.63
**SVA-13_4**	Vestre Brøggerbreen	20/07/2013	33H 0432976 E, 8760004 N	254	6.10	0	39			0.67
**SVA-13_10**	Midtre Lovénbreen	21/07/2013	33H 0436410 E, 8757512 N	299	6.65	0	54	7.49	3.99	0.76
**SVA-13_20**	Austre Brøggerbreen	24/07/2013	33X 0429286 E, 8761458 N	209	6.38		91			0.60
**SVA-13_23**	Austre Brøggerbreen	24/07/2013	33X 0429448 E, 8761568 N	227	6.27		75			0.63
**SVA-13_31**	Austre Brøggerbreen	24/07/2013	33X 0430139 E, 8761706 N	146	8.07		70			0.62
**SVA-13_33**	Pedersenbreen	27/07/2013	33X 0441747 E, 8756068 N	320			22	24.7	1.07	0.55
**SVA-13_36**	Pedersenbreen	27/07/2013	33X 0441609 E, 8756682 N	262			115			0.55
**SVA-13_43**	Austre Lovénbreen	03/08/2013	33X 0439635 E, 8756676 N	413	6.3	0.1	182	19.6	0.66	0.66
**SVA-13_48**	Austre Lovénbreen	03/08/2013	33X 0438286 E, 8756948 N	345			36			0.60
**SVA-13_54**	Feiringsbreen	05/08/2013	33X 0446691 E, 8773282 N	401	5.68		88	11.8	0.35	0.51
**SVA-13_65**	Midtre Lovénbreen	05/08/2013	33X 0436693 E, 8759332 N	151			108			0.49
**Tarfala, Sweden**										
**TAR-13_1**	Storglaciären	01/07/2013	34W 0398931 E, 7533637 N	1268	6.98	0	52	8.05	0.08	0.56
**TAR-13_5**	Storglaciären	01/07/2013	34W 0399260 E, 7534131 N	1221	5.56	0	30			0.66
**TAR-13_8**	Storglaciären	03/07/2013	34W 0397551 E, 7534187 N	1412	7.23	0.1	97			0.72
**TAR-13_17**	Rabot	05/07/2013	34W 0394929 E, 7534801 N	1350	6.13	0	112	16.5	1.05	0.75
**TAR-13_21**	Rabot	05/07/2013	34W 0394160 E, 7535197 N	1282	5.45	0	122			0.73
**TAR-13_24**	Rabot	05/07/2013	34W 0393074 E, 7534485 N	1165			95			0.54
**TAR-13_27**	Liljetopsrännan	06/07/2013	34W 0398423 E, 7533989 N	1119	5.41	0.2	54	11.1	0.4	0.65
**TAR-13_28**	Liljetopsrännan	06/07/2013	34W 0398656 E, 7536883 N	1209	6.35	0	51			0.56
**TAR-13_30**	SE-Kasskasatjåkkå	07/07/2013	34W 0399446 E, 7537111 N	1374	0.45	0.3	123	19.4	2.78	0.77
**TAR-13_32**	SE-Kasskasatjåkkå	07/07/2013	34W 0399458 E, 7536982 N	1318	5.78	0	108			0.57
**TAR-13_35**	Storglaciären	09/07/2013	34W 0398849 E, 7534337 N	1308	5.41	0	88			0.76
**TAR-13_36**	Permanent snow field	09/07/2013	34W 0399453 E, 7534692 N	1167	5.68	0	85			0.65
**TAR-13_37**	Permanent snow field	09/07/2013	34W 0399376 E, 7534942 N	1163	5.73	0.4	82			0.62
**TAR-13_39**	Permanent snow field	10/07/2013	34W 0400256 E, 7535905 N	1318			199	18.4	3.86	0.66
**TAR-13_41**	Björling	11/07/2013	34W 0395764 E, 7532198 N	1295	5.44	0	127	20.3	4.49	0.66
**TAR-13_42**	Björling	11/07/2013	34W 0396623 E, 7531127 N	1156	6.1	0.3	100			0.57
**TAR-14_1**	Storglaciären	04/07/2014	34W 0398031 E, 7533618 N	1268	7.4	0.1				
**TAR-14_4**	Storglaciären	07/07/2014	34W 0398886 E, 7533623 N	1277	7.35	0.1				
**TAR-14_5**	Storglaciären	07/07/2014	34H 0399085 E, 7533632 N	1247	7.78	0.3				
**TAR-14_6**	Storglaciären	07/07/2014	34H 0393734 E, 7535101 N	1226	6.17	0.1				
**TAR-14_7**	Rabot	09/07/2014	34W 0394036 E, 7535053 N	1326	8.13	0.1				

**TAR-14_10**	Liljetopsrännan	10/07/2014	34W 0398650 E, 7536880 N	1215	7.51	0.1				
**TAR-14_11**	SE-Kasskasatjåkkå	10/07/2014	34W 0399438 E, 7537029 N	1340	7.14	0.1				
**TAR-14_12**	Permanent snow field	10/07/2014	34W 0400194 E, 7535878 N	1318	8.16	0.1				
**Mittivakkat, Greenland**										
**MIT-12_7**	Mittivakkat	10/07/2012	24H 0551567 E, 7285460 N	155	5.67	0	204	28.1	1.13	
**MIT-12_19**	Mittivakkat	17/07/2012	24H 0551778 E, 7286368 N	150	4.68	0.73	345	46.2	1.54	39
**Iceland**										
**ICE-12_3**	Drangajökull	27/07/2012	27W 0442125 E, 7334250 N	196	6.15					
**ICE-12_4**	Laugafell	29/07/2012	27W 0632892 E, 7222179 N	908	4.96		237	21.2	1.24	42

Supplementary Table 2: Bacterial community composition

Distribution of 97% clustered OTUs aligned and assigned to known bacterial species. Values are the relative abundance of the taxa in percentage of total sequences and figure shows taxa with >0.01% abundance. It is important to note that values are rounded to one digit; therefore the abundance of a taxon with a value of 0 in one sample can range between 0 and 0.04%.

**Phylum**	***Acidobacteria***		***Actinobacteria***		***Bacteriodetes***				***Chlorobi***	***Chloroflexi***		
**Class**	**Acidobacteriia**	***Solibacteres***	***Acidimicrobiia***	***Actinobacteria***	***Cytophagia***	***Flavobacteriia***	***Sphingobacteriia***	***Saprospirae***	***Ignavibacteria***	***Anaerolineae***	***C0119***	***Ktedonobacteria***
**SVA-13_4**	0	0	0	0.5	37.4	0	0.3	51.2	0	0	0	0
**SVA-13_10**	17	0	0	0	9	0	0	3	0	0	0	0
**SVA-13_20**	0	0	0	0	10.6	0	1.5	45.2	1.8	0	0	0
**SVA-13_23**	0	0	0	0.3	6.3	4.6	0	1	0	0	0	0
**SVA-13_31**	0	0	0	4.2	11.6	0.4	0	44.9	0	0	0	0
**SVA-13_33**	0.4	0	0	2.1	3.9	0	2.8	26.8	0	0	0	0
**SVA-13_36**	3.4	0	0	8.4	39	0.5	0.8	1.6	0	0	0	0
**SVA-13_43**	0.8	0	0	0.8	12.5	0	0.4	67.3	0	0	0	0
**SVA-13_48**	0	0	0	0.6	44.5	0.6	0	19.1	0	0	0	0
**SVA-13_54**	0.2	0	0	15.7	3.4	0.5	34.6	3.6	0	0	0	0
**SVA-13_65**	0.4	0	0	0.2	5.9	1	0.3	3.7	0	0.1	0	0
**TAR-13_1**	0	0	0	0.1	3.6	0	91.1	1.9	0	0	0	0
**TAR-13_8**	0.4	0	0	0	0	0	89.3	0	0.4	0	0	0
**TAR-13_17**	0.2	0	0	0	0.8	0	90.7	0.1	0	0	0	0
**TAR-13_21**	0	0	0	0	1	0	75.7	22.3	0	0	0	0
**TAR-13_27**	0	0	0	0	0.5	0	71.4	0.5	5.1	0	0	0
**TAR-13_28**	4.7	0	0	0	0	0	12.5	0	0	0	0	0
**TAR-13_30**	0.2	0	0	0	3.8	0	81.6	3.2	0.2	0	0	0
**TAR-13_35**	0.8	0	0	0.1	4.1	0.9	63	4.3	0	0	0	0
**TAR-13_41**	0	0	0	0.1	0.5	0	82.1	15.8	0.3	0	0	0
**TAR-14_1**	1.1	0	0	4.3	1.2	0	46.3	11.2	0	0	0	0
**TAR-14_4**	10.7	0.3	0	0	0	0	0.2	0	0	0.1	0	0
**TAR-14_5**	16.7	0.2	0	0	15	0	1.3	0	0	0	0	0
**TAR-14_6**	0.1	0	0	0.1	1.9	0.1	81.3	2.4	0	0	0	0
**TAR-14_7**	17.2	0.3	0	0	0	0.1	1.7	0	0	0	0	0
**TAR-14_10**	17.1	0.7	0	0.1	7.3	0	45.8	1	0	0	0	0
**TAR-14_11**	4.6	0.5	0	0.4	13.6	0	51.8	6.3	0	0	0	0
**TAR-14_12**	3.1	0	0	0.3	2.2	0	59.3	0	0	0	0	0
**MIT-12_7**	1.2	0	0	0.3	4.8	0	33.2	12.4	0	0	0	0
**MIT-12_19**	0	0	0	1.1	2.9	0	14.6	10.8	0	0	0	0
**ICE-12_3**	0.4	0	0	6.0	0.1	0	18.8	24.1	0	0	0	0
**ICE-12_4**	0	0	0	2.6	5.4	0	0	28.5	0	0	0	0

Supplementary Table 2 continued.

**Phylum**	***Cyanobacteria***				***Fibrobacteres***	***Firmicutes***	***Gemmatimonadetes***	***Proteobacteria***				***TM7-3***	***WPS-2***	***Thermi***
**Class**	**Cyano-bacteria**	**Nostoco-phycideae**	**Oscillatorio-phycideae**	**Synechococco-phycideae**	**Fibrobacteria**	**Clostridia**	**Gemmatimonadetes**	**Alphaproteo-bacteria**	**Betaproteo-bacteria**	**Deltaproteo-bacteria**	**Gammaproteo-bacteria**	**TM7-3**	**WPS-2**	**Deinococci**
**SVA-13_4**	0	0.3	0	0.5	0	0	0	0	9.5	0	0.3	0	0	0
**SVA-13_10**	0	1	17	20	0	2	0	8	22	0	0	0	1	0
**SVA-13_20**	0	0.6	1.2	1.5	0	0	0	0	30.2	0	7.6	0	0	0
**SVA-13_23**	0.3	2.9	0.1	77.3	0	1.3	0	0.7	4.9	0	0.3	0	0	0
**SVA-13_31**	0	1.8	0.4	10.2	0	0	0	1.1	25.6	0	0	0	0	0
**SVA-13_33**	0	0.2	0	0.6	0	0.2	0	2.1	60.8	0	0	0	0	0
**SVA-13_36**	0	0	0	8.2	0	0	0	12	24.3	0.1	0	0.1	0	1.4
**SVA-13_43**	0	0	0	0	0	0	0	0.8	17.5	0	0	0	0	0
**SVA-13_44**	0	0	0	0.1	0	0	0	0.8	49.4	0	0	0	0	0
**SVA-13_47**	0	0	0	43.6	0	0	0.1	11.3	0.6	0.2	0	0.3	3.3	0
**SVA-13_48**	0	0	0	4.2	0	0	0	0.6	30.4	0	0	0	0	0
**SVA-13_54**	0	0.2	0	18.5	0	0	0	4.8	18.4	0	0.2	0	0	0
**SVA-13_65**	0	0.2	0	24.2	0	0.1	0	0.9	62.6	0	0.4	0	0.1	0
**TAR-13_1**	0.1	0	0	0.1	0	0	0	0.1	2.1	0	1	0	0	0
**TAR-13_8**	0.8	3.1	3.3	0	0	0	0	2.3	0.4	0	0	0	0.1	0
**TAR-13_17**	0.2	4.8	1.4	0.1	0	0	0	1.3	0.4	0	0	0	0	0
**TAR-13_21**	0.1	0	0	0.1	0	0	0	0.1	0.3	0	0.2	0	0	0
**TAR-13_27**	0	4.3	0.8	0.5	0	0	0	6.6	0.3	0	10	0	0	0
**TAR-13_28**	1.6	20.3	4.7	6.3	0	0	0	50	0	0	0	0	0	0
**TAR-13_30**	0.7	1.1	0.9	0.1	0	0	0	1.1	7	0	0.2	0	0	0
**TAR-13_35**	0	3	0.4	20.6	0	0.2	0	1	1.3	0	0.4	0	0	0
**TAR-13_41**	0	0.2	0.2	0.2	0	0	0	0.5	0.1	0	0	0	0	0
**TAR-14_1**	0	0.4	0.4	1.8	0	0	0	18.7	14.4	0	0.2	0	0	0
**TAR-14_4**	0.6	38.6	30	5	0	0	0	13.9	0.5	0	0	0	0	0
**TAR-14_5**	3	12.7	15.5	1.9	0	0	0	32.2	1.3	0	0	0	0.2	0
**TAR-14_6**	0	0.1	0.1	10.2	0	0	0	2.1	1.1	0	0.5	0	0	0
**TAR-14_7**	3.1	42.8	2.5	1.8	0	0	0	26.1	4.4	0	0	0	0.1	0
**TAR-14_10**	0.2	0.7	0.3	0.5	0	0	0	8.8	16.9	0	0.4	0	0.2	0
**TAR-14_11**	0	0.1	0.2	0.1	0	0	0	4.4	4.2	0	13.8	0	0.2	0
**TAR-14_12**	0	0.6	0	16.7	0	0	0	17	0.3	0	0.6	0	0	0
**MIT-12_7**	0	0.6	0.3	0	0	0	0	1.2	45.3	0	0.6	0	0	0
**MIT-12_19**	0	50.2	7	2.5	0	0	0	8.2	2.7	0	0	0	0	0
**ICE-12_3**	0	0	0	0.5	0	0	0.1	42.0	4.9	0	1.3	0	0.9	0
**ICE-12_4**	0	0.2	0	0.5	0	0	0.5	5.4	56.2	0	0	0	0	0

Supplementary Table 3: Measured average values and statistics of all analyses

Statistical analysis of all biological and geochemical results analysed by one-way ANOVA and post-hoc Tukey's test to reveal significant differences between the red snow samples of the sampled geographic locations Svalbard (SVA), Northern Sweden (TAR), Greenland (MIT) and Iceland (ICE). Table shows mean values for each geographic location with respective standard deviations and p-values for the overall significance of the differences between all locations as well as post-hoc pair-wise comparison between two locations (e.g., SVA x TAR). Results with p-values of <0.05 were considered to be significant and are in bold, results with p-values <0.01 were considered to be highly significant and are also underlined.

	All locations	SVA Measured average values	SVA x TAR p value	TAR Measured average values	TAR x MIT p value	MIT Measured average values	MIT x SVA p value	ICE Measured average values	ICE x SVA p value	ICE x TAR p value	ICE x MIT p value
**Algae**											
Chloromonas alpina **[%]**	0.858	0.48 ± 0.64	0.870	1.22 ± 3.42	0.938	0.12 ± 0.16	0.998	0.93 ± 1.05	0.996	0.999	0.999
Chloromonas nivalis **[%]**	**0.010**	3.07± 4.25	0.790	4.56 ± 3.80	0.057	12.99 ± 6.75	**0.022**	10.78 ± 5.81	0.100	0.220	0.952
Chloromonas polyptera **[%]**	0.190	9.63 ± 6.24	0.601	14.47 ± 12.62	0.970	17.86 ± 7.57	0.715	25.59 ± 3.20	0.191	0.474	0.871
Raphidonema sempervirens **[%]**	0.095	5.89 ± 11.06	0.758	2.81 ± 5.87	0.994	1.23 ± 1.74	0.878	18.32 ± 2.34	0.215	0.077	0.181
**Uncultured *Chlamydomonadaceae* (a) [%]**	**0.030**	74.91 ± 14.84	0.590	67.62 ± 16.06	0.961	62.12 ± 1.47	0.685	38.92 ± 6.24	**0.020**	0.075	0.426
**Uncultured *Chlamydomonadaceae* (b) [%]**	0.322	4.83 ± 3.44	0.998	5.09 ± 3.90	1.000	4.97 ± 0.88	1.000	0 ± 0	0.311	0.254	0.518
**Bacteria**											
Bacteriodetes **[%]**											
Sphingobacteria **[%]**	**<0.001**	3.68 ± 10.29	**<0.001**	60.20 ± 28.56	0.159	23.94 ± 13.15	0.641	9.42 ± 13.31	0.987	0.026	0.913
Saprospirae **[%]**	**0.023**	24.31 ± 24.15	**0.020**	4.57 ± 6.77	0.933	11.62 ± 1.1	0.725	26.28 ± 3.1	0.998	0.284	0.79
Cytophagia **[%]**	0.059	16.72 ± 15.5	0.060	5.24 ± 6.90	0.998	3.84 ± 1.4	0.430	2.74 ± 3.78	0.359	0.990	1
Cyanobacteria **[%]**											
Nostocophycideae**[%]**	**0.005**	0.64 ± 0.92	0.826	3.39 ± 5.73	**0.006**	25.40 ± 35.07	0.003	0.12 ± 0.17	1.000	0.949	0.021
Oscillatoriophycideae **[%]**	0.872	1.69 ± 5.09	1.000	1.87 ± 4.00	0.948	3.67 ± 4.76	0.937	0 ± 0	0.959	0.942	0.839
Synechococcophycideae **[%]**	0.384	15.02 ± 22.40	0.497	6.14 ± 10.04	0.976	1.24 ± 1.75	0.669	0.49 ± 0.03	0.631	0.964	1
Proteobacteria**[%]**											
Alphproteobacteria **[%]**	0.144	2.82 ± 3.90	0.489	9.67 ± 14.42	0.946	4.69 ± 4.92	0.997	23.73 ± 25.90	0.133	0.422	0.405
Betaproteobacteria **[%]**	**0.002**	27.84 ± 18.48	**0.002**	3.36 ± 5.36	0.297	23.99 ± 30.17	0.987	30.57 ± 36.31	0.995	0.108	0.973
**Diversity**											
**Shannon eukaryotes**	0.103	4.51 ± 1.29	0.964	4.66 ± 0.84	0.230	6.13 ± 0.02	0.166	5.79 ± 0.33	0.355	0.463	0.985
**Shannon algae**	0.126	3.06 ± 1.17	0.847	3.34 ± 0.82	0.536	4.27 ± 0.24	0.331	4.49 ± 0.02	0.204	0.362	0.996
**Shannon bacteria**	0.130	5.67 ± 1.04	0.191	4.95 ± 1.00	0.309	6.19 ± 0.38	0.902	5.20 ± 0.10	0.923	0.974	0.748

**Solids (C,N,P,S)**											
**TC [%]**	**0.007**	5.4 ± 4.4	**0.007**	12.4 ± 7.2							
**TN [%]**	**0.047**	0.3 ± 0.2	**0.047**	0.6 ± 0.4							
**TS [%]**	**<0.001**	0.12 ± 0.03	**<0.001**	0.07 ± 0.02							
**TP [%]**	0.153	0.07 ± 0.03	0.153	0.06 ± 0.01							
**C/N [ ]**	**0.009**	16 ± 3	**0.009**	21 ± 6							
**C/P [ ]**	**<0.001**	65 ± 31	**<0.001**	186 ± 89							
**N/P [ ]**	**0.001**	4 ± 2	**0.001**	8 ± 3							
**$d^{15}N$ [‰]**	0.582	-4.77 ± 2.41	0.582	-4.30 ± 1.84							
**$d^{13}C$ [‰]**	**0.033**	-27.76 ± 1.52	**0.033**	-25.70 ± 2.02							
**Metabolites**											
**Secondary carotenoids [%]**	0.722	80 ± 7	0.722	77 ± 19							
**Saturated fatty acids [%]**	0.218	32 ± 5	0.218	37 ± 14							
**Monounsaturated fatty acids[%]**	0.173	16 ± 5	0.173	13 ± 4							
**Polyunsaturated fatty acids [%]**	0.449	50 ± 8	0.449	46 ± 13							
**Biomass**											
**Cell counts [mL^{-1}]**	0.083	43,558 ± 50,308	0.083	12,121 ± 10,552							
**Cell volume [μm^3]**	0.305	2,044 ± 1,342	0.305	2,859 ± 1,381							
**Biomass [mm^3 L^{-1}]**	0.263	35 ± 20	0.263	20 ± 15							
**Aqueous**											
**DOC [µM]**	**<0.001**	36 ± 13	**<0.001**	189 ± 90							
**PO4 [uM]**	0.736	0.77 ± 1.71	0.736	0.54 ± 1.08							
**NO3 [ppm]**	0.399	1008 ± 1986	0.414	236 ± 623	0.996	27 ± 37	0.760	**<0.001**	0.745	0.995	1
**SO4 [ppm]**	**0.013**	13 ± 46	0.981	17 ± 49	**0.007**	197 ± 278	**0.007**	91 ± 128	0.522	0.549	0.482
**Ca [ppb]**	0.121	381 ± 588	0.106	45 ± 66	1.000	19 ± 1	0.583	24 ± 3	0.595	1	1
**Fe [ppb]**	**<0.001**	1.9 ± 1.0	0.983	1.6 ± 1.0	0.175	5.0 ± 1.4	0.256	10 ± 10	**<0.001**	**<0.001**	0.110
**K [ppb]**	0.395	41 ± 20	0.583	77 ± 92	0.851	121 ± 129	0.492	41 ± 4	1.000	0.913	0.695
**Mg [ppb]**	0.116	125 ± 206	0.092	7 ± 4	1.000	5 ± 3	0.621	39 ± 36	0.665	1	1
**Mn [ppb]**	**0.008**	3.7 ± 3.9	**0.005**	0.3 ± 0.3	0.977	1 ± 0	0.470	0.7 ± 1.0	0.378	0.995	0.999

Supplementary Table 4: Algal community composition

Distribution of 97% clustered OTUs aligned and assigned to *Archaeplastida* (green algae). Values are the relative abundance of the taxa in percentage of total sequences and table shows taxa with OTUs of a minimum total observation count of 0.1%. It is important to note that values are rounded to one digit; therefore the abundance of a taxon with a value of 0 in one sample can range between 0 and 0.04%.

Species	*Ancylonema nordenskioeldii*	*Chloromonas cf. alpina* AF514403	*Chlamydomonas cf. proboscigera*	*Chloromonas nivalis* AF514409	*Chloromonas polyptera* JQ790556	*Chloromonas spec. CCCryo 273-06*	*Chloromonas tughillensis*	*Microglena polar subclade Chlamydo-Monas* EF537906	*Prototheca cutis*	*Raphidonema sempervirens* AJ309939	*Trebouxia usneae*	Uncultured *Chlamydomonadaceae* (1) GU117577	Uncultured *Chlamydomonadaceae* (2) GU117576	Uncultured *Chlamydomonadaceae* (3) GU117575
**SVA-13_2**	0	0.1	0	1.8	22.5	0	0	0	0	0	0.2	72.3	3	0.2
**SVA-13_4**	0	0.8	0	0.7	0.9	0	0.9	0.1	0	0	0	87.8	8.8	0
**SVA-13_10**	0	0	0	0.2	5.3	0	0	0	0	0	0	83.8	10.7	0.1
**SVA-13_20**	0	0	0	14	8.8	0	0.4	0	0	1.7	0	65	10.1	0
**SVA-13_23**	0	0	0	0.6	6.5	0	0	0	0	0.2	0	89.9	2.6	0.1
**SVA-13_31**	0	0	0	2.7	5.8	0	0	0	0.3	0.5	0	88.9	1.8	0
**SVA-13_33**	0	1.9	0	9.3	10.8	0.3	0	0	0	5.1	0	65.2	7.6	0
**SVA-13_36**	8.6	0.3	0.1	1.3	16.5	0	0.1	0	0.3	3.6	0	65.9	3.2	0.1
**SVA-13_43**	0	0.9	0	2.8	10.3	0	0.3	0.1	0	35.5	0	45.8	4.2	0.1
**SVA-13_48**	0	1.4	0	2.8	12.4	0	0	0	0.1	21.3	0	59.3	2.1	0.5
**SVA-13_54**	0	0	0	0.6	1.5	0.1	0	0	0	2.3	0	93.3	2.1	0
**SVA-13_65**	0.1	0.2	1.2	0	14.3	0	0	0	0.1	0.5	0	81.8	1.8	0.1
**TAR-13_1**	0	0	0	1.2	12.2	0	1.3	0	0	0	0.1	76.1	8.9	0
**TAR-13_8**	0	5.7	0	4.6	52.6	0	0.1	0	0.1	0.1	0	33.4	3.3	0
**TAR-13_17**	0	0.2	0	0.4	9	0	0	0	0	14.1	0	67.7	8.6	0
**TAR-13_21**	0	0.1	0	12.6	6.1	0	1.3	0	0	0	0	72.1	7.6	0.1
**TAR-13_27**	0	0	0	8.8	17	0	0.1	0	0.1	1.7	0.1	69.0	3.2	0
**TAR-13_28**	0	0	0	1.1	17.6	0	0	0	0	0.3	0	74.5	6.5	0
**TAR-13_30**	0	0	0	2	1	0	0.3	0.6	0	1.9	0.1	87.7	6.4	0
**TAR-13_35**	0	12.9	0	7.2	26.9	0	0.9	0	0.4	0	0.1	32.1	0.7	18.9
**TAR-13_39**	0	0	0	5.2	6.4	0	0.1	0	0	0	0	75.3	13	0
**TAR-13_41**	0	0	0	8.3	5.4	0	2.7	0	0	0.1	0	71.8	11.6	0
**TAR-14_1**	0	0.3	0	0.7	23.2	0	0.1	0	16.8	0.7	0.2	54.8	1.9	1.4
**TAR-14_4**	0	1	0	3.3	10.1	0	0.4	0	0.5	1	12.2	68.9	2.4	0.1
**TAR-14_5**	0	0.1	0	2.7	4.9	0	0.2	0	0.2	0.1	5.1	84.1	2.7	0
**TAR-14_6**	0	0	0	0	8.1	0	0	0	4.4	0.5	0	85.1	2	0
**TAR-14_7**	0	22.7	0	0	53.1	0	0	0	1.5	0.4	10	7.9	0.4	4
**TAR-14_10**	0.2	0	0	9.4	14.4	0	0.1	0	0.3	1.2	0.9	71.1	2.4	0
**TAR-14_11**	0.2	0	0	5.1	21.1	0	0	0	0.2	3.6	8.5	59.8	1.5	0
**TAR-14_12**	0	0	0	3.8	5.7	0	0	0	0	20.5	1.2	67.3	1.3	0.2
**MIT-12_7**	0	0	0	17.8	12.5	0	1	0	0	0	0	63.2	5.6	0
**MIT-12_19**	0	0.2	0	8.2	23.2	0	0.4	0	0	2.5	0.1	61.1	4.3	0
**ICE-12_3**	0	0.2	0	14.9	27.8	0	4.6	0	0	16.6	0	34.5	1.4	0
**ICE-12_4**	0	1.7	0	6.7	23.3	0	1.7	0	0	20.0	0	43.3	3.3	0

Supplementary Table 5: Eukaryotic community composition

Distribution of 97% clustered OTUs aligned and assigned to eukaryotes. Values are the relative abundance of the taxa in percentage of total sequences. It is important to note that values are rounded to one digit; therefore the abundance of a taxon with a value of 0 in one sample can range between 0 and 0.04%.

Taxon	*Amoebozoa*	*Archaeplastida*	*Centrohelida*	*Kathablepharidae*	*Opisthokonta*	*RT5iin25*	*SAR*	*Zeuk77*
**SVA-13_2**	0	68.0	0	0	31.5	0	0.5	0
**SVA-13_4**	0	83.3	0	0	4.0	0	12.7	0.0
**SVA-13_10**	0	74.5	0	0	24.7	0	0.7	0
**SVA-13_20**	0	81.8	0	0	12.9	0	5.3	0
**SVA-13_23**	0.0	63.3	0	0	32.5	0	2.9	0
**SVA-13_31**	0.0	82.4	0	0	9.1	0	7.9	0
**SVA-13_33**	0	58.0	0	0	26.8	0	14.2	1.0
**SVA-13_36**	0	42.0	0	0	38.4	0	19.0	0.1
**SVA-13_43**	0	56.6	0	0	24.4	0	17.5	1.5
**SVA-13_48**	0.1	82.5	0	0	12.9	0	4.2	0
**SVA-13_54**	0	62.3	0	0	33.8	0	3.8	0
**SVA-13_65**	0	67.8	0	0	20.8	0	9.3	0
**TAR-13_1**	0	64.1	0	0	32.1	0	3.8	0
**TAR-13_8**	0	44.7	0	0	47.9	0	7.4	0
**TAR-13_17**	0	27.1	0	0	72.6	0	0.3	0
**TAR-13_21**	0	68.0	0	0	23.4	0	8.5	0.1
**TAR-13_27**	0	29.5	0	0	68.3	0	1.8	0
**TAR-13_28**	0	76.4	0	0	23.2	0	0.4	0
**TAR-13_30**	0	38.1	0	0	61.0	0	1.0	0
**TAR-13_35**	0	72.8	0	0	19.6	0	6.7	0
**TAR-13_39**	0	88.0	0	0	11.0	0	1.0	0
**TAR-13_41**	0	75.0	0	0	19.8	0	5.2	0
**TAR-14_1**	0.2	38.1	0	0	50.9	0	10.4	0
**TAR-14_4**	0	14.2	0	0	69.9	0	15.3	0
**TAR-14_5**	0	32.6	0	0	58.5	0	8.6	0
**TAR-14_6**	0.1	37.2	0	0	51.1	0	9.7	0
**TAR-14_7**	0	47.6	0	0	48.4	0	1.4	0
**TAR-14_10**	0	26.0	0	0	71.8	0	1.4	0
**TAR-14_11**	0	18.2	0	0	77.2	0	3.8	0
**TAR-14_12**	0	68.8	0	0	21.1	0	9.5	0.2
**MIT-12_7**	0	47.4	0	0	22.9	0	29.7	0
**MIT-12_19**	0	53.0	0	0	25.8	0	21.2	0
**ICE-12_3**	0	45.3	0	0	23.2	0	30.7	0.1

**ICE-12_4**	0	19.8	0	0	29.0	0	49.6	1.6

Supplementary Table 6: “SAR” community composition

Distribution of 97% clustered OTUs aligned and assigned to the “SAR”-group (*stramenopiles, alveolata, rhizaria*) and taxa within the *stramenopiles*. Values are the relative abundance of the taxa in percentage of total. The “SAR”-group was screened in order to screen for other other pigmented micro-eukaryotes, *i.e*., the *Chrysophyceae*. In nearly all samples the relative abundance of *Chryosphyceae* was below 0.1% and therefore they are considered negligible in terms of contribution to albedo.

	Alveolata	***Rhizaria***	***Stramenopiles***	***Stramenopiles***									
				CCI40	*Chrysophyceae*						*Peronosporomycetes*	*Synurales*	*Xanthophyceae*
					CCMP1899, Chrysophyceae sp. I76	*CCMP1899, Ochromonas*	*CCMP1899*	*Chromulinales, LG31-02*	*Chrysocapsales, Hydrurus*	*Ochromonadales, Paraphysomonas*	*Phytophthora, Halophytophthora*	*Synura, Synura uvella*	*Tribonematales, Botrydiopsis*
**SVA-13_2**	0.2	0.3	0	0	0	0	0	0	0	0	0	0	0
**SVA-13_4**	1.6	11.0	0.0	0	0	0	0	0	0	0	0.03	0	0
**SVA-13_10**	0.2	0.5	0.1	0	0	0.02	0.03	0	0	0	0	0	0
**SVA-13_20**	0.3	5.1	0	0	0	0	0	0	0	0	0	0	0
**SVA-13_23**	0.0	2.9	0	0	0	0	0	0	0	0	0	0	0
**SVA-13_31**	0.1	5.2	2.6	0	0.01	0.05	2.46	0.06	0	0	0	0	0
**SVA-13_33**	6.6	7.4	0.1	0.03	0	0	0.03	0	0	0	0	0	0
**SVA-13_36**	4.6	14.3	0.1	0	0	0	0.01	0	0	0.02	0	0	0.01
**SVA-13_43**	7.2	10.3	0	0	0	0	0	0	0	0	0	0	0
**SVA-13_48**	0.0	3.4	0.7	0	0.02	0	0.7	0.02	0	0	0	0	0
**SVA-13_54**	0.8	2.4	0.1	0	0	0	0.08	0	0	0	0	0	0.05
**SVA-13_65**	0.5	5.0	3.8	0	0.02	0.06	3.74	0	0	0	0	0	0
**TAR-13_1**	0.1	3.7	0	0	0	0	0	0	0	0	0	0	0
**TAR-13_8**	1.4	6.0	0	0	0	0	0	0	0	0	0	0	0
**TAR-13_17**	0.1	0.3	0	0	0	0	0	0	0	0	0	0	0
**TAR-13_21**	0.4	7.9	0.2	0	0	0	0	0	0	0	0.23	0	0
**TAR-13_27**	0.0	1.8	0.0	0	0	0	0	0	0.02	0	0	0	0
**TAR-13_28**	0.0	0.4	0	0	0	0	0	0	0	0	0	0	0
**TAR-13_30**	0.0	1.0	0	0	0	0	0	0	0	0	0	0	0
**TAR-13_35**	4.8	1.9	0.0	0	0	0	0	0	0.02	0	0	0	0
**TAR-13_39**	0.1	1.0	0	0	0	0	0	0	0	0	0	0	0
**TAR-13_41**	0.1	5.2	0	0	0	0	0	0	0	0	0	0	0
**TAR-14_1**	0.0	10.3	0.1	0	0	0	0	0	0.02	0	0	0	0
**TAR-14_4**	0.0	15.2	0.0	0	0	0	0	0	0.01	0	0	0	0

**TAR-14_5**	0.1	8.5	0.1	0	0	0	0	0	0.06	0	0	0	0
**TAR-14_6**	0.1	9.7	0	0	0	0	0	0	0	0	0	0	0
**TAR-14_7**	0	1.43	0	0	0	0	0	0	0	0	0	0	0
**TAR-14_10**	0.1	1.2	0.1	0	0	0	0	0	0.13	0	0	0	0
**TAR-14_11**	0.5	3.2	0.2	0	0	0	0	0	0.12	0	0	0.03	0
**TAR-14_12**	0.8	1.7	7.0	0.07	0	0	0	0	6.79	0	0	0.11	0
**MIT-12_7**	1.7	28.0	0	0	0	0	0	0	0	0	0	0	0
**MIT-12_19**	0.8	20.4	0	0	0	0	0	0	0	0	0	0	0
**ICE-12_3**	0.5	30.11	0.1	0	0	0	0	0	0.11	0	0	0	0
**ICE-12_4**	6.4	42.46	0.8	0	0	0	0	0.40	0.20	0	0	0	0.20

Supplementary Table 7: Archaeal community composition

Distribution of 97% clustered OTUs aligned and assigned to archaea. It is important to note that values are rounded to one digit; therefore the abundance of a taxon with a value of 0 in one sample can range between 0 and 0.04%. The archaeal community composition revealed no biogeographical patterns and was also characterised by low species diversity. In most samples the main representatives were *Crenarchaeota* with the order *Nitrososphaerales* dominating (up to 100%).

**Phylum**	***Crenarchaeota***					***Euryarchaeota***			
**Class**<br>**Order**	***MBGA***	***MBGA; NRP-J***	***MCG; pGrfC26***	***Thaumarchaeota; Cenarchaeales***	***Thaumarchaeota; Nitrososphaerales***	***Methanobacteria; Methanobacteriales***	***Methanomicrobia; Methanomicrobiales***	***Methanomicrobia; Methanosarcinales***	***Thermoplasmata; E2***
**SVA-13_2**	0	0	0	0.3	99.7	0.0	0	0	0
**SVA-13_23**	0	0	0	0.5	99.4	0.2	0	0	0
**SVA-13_31**	0	0	0	0.0	99.4	0.6	0	0	0
**SVA-13_36**	0	0	0	3.6	92.9	2.4	0	0	1.2
**SVA-13_48**	0	0	0	5.0	16.8	78.2	0	0	0
**SVA-13_54**	0	0	0	13.2	74.5	8.5	0	0	3.8
**SVA-13_65**	0	0	0	14.3	42.9	28.6	14.3	0	0
**TAR-13_8**	0	0	0	4.9	86.2	2.0	1.2	0	5.7
**TAR-14_1**	0	0	0	0.1	100.0	0	0	0	0
**TAR-14_4**	0	0	0	0.6	99.4	0	0	0	0.0
**TAR-14_5**	0	0	0	2.2	97.8	0	0	0	0
**TAR-14_6**	0	0	0	0	0	0	0	90.0	10.0
**ICE-12_3**	0	0	0	2.8	96.5	0	0	0	0
**ICE-12_4**	0	0	0	0.7	27.6	0	0	71.6	0

Supplementary Table 8: Quality control of DNA sequences

Number of sequences before and after quality control, assigned to taxa and with respective Shannon diversity indices for eukaryotes, algae and bacteria, which did not show significant differences between locations (Supplementary Table 14). Shannon indices for archaea have not been calculated due to the very low species diversity.

	Eukaryotes			Algae		Bacteria				Archaea		
	**Raw seqs**	**Seqs after QC**	**Shannon**	**Seqs assigned to algae**	**Shannon**	**Raw seqs**	**Seqs after QC**	**Seqs assigned to bacteria**	**Shannon**	**Raw seqs**	**Seqs after QC**	**Seqs assigned to archaea**
**SVA-13_2**	6135	5148	3.34	3500	2.34	98*	4*	1*		60965	54688	53817
**SVA-13_4**	6979	3937	4.56	3280	3.41	7509	1421	770	6.16			
**SVA-13_10**	13448	6583	4.24	4904	3.52	29728	3511	334	6.69			
**SVA-13_20**	7130	3952	5.20	3226	4.08	12881	2344	987	7.00			
**SVA-13_23**	5373	4485	2.93	2832	1.74	8352	4008	1850	5.36	1695	732	657
**SVA-13_31**	12752	10621	3.07	8743	1.79	5040	2341	598	5.52		43331	42417
**SVA-13_33**	7678	3008	6.22	1727	4.40	9129	1911	1041	6.86			
**SVA-13_36**	23283	16789	5.28	7056	2.93	11503	6109	2573	4.26	2318	921	272
**SVA-13_43**	16457	5447	7.24	3059	5.43	5473	988	627	6.60			
**SVA-13_48**	6294	5123	3.96	4184	2.93	2838	1183	556	4.83	2665	722	410
**SVA-13_54**	23068	16698	3.89	10391	1.68	3777	1519	812	4.84	39830	34942	34121
**SVA-13_65**	7362	5408	4.17	3667	2.42	13545	7349	4245	4.25	1267	295	68
**TAR-13_1**	9632	4770	5.29	3058	4.23	21581	6431	5087	4.39			
**TAR-13_8**	77757	26947	5.85	12012	4.64	12368	3661	1051	4.39	2059	926	310
**TAR-13_17**	8767	4863	4.85	1318	4.68	16240	5850	3632	4.36			
**TAR-13_21**	9330	4354	5.25	2920	3.89	10946	3997	3169	5.45			
**TAR-13_27**	19416	15725	2.55	4639	3.04	16105	2297	630	5.87			
**TAR-13_28**	9402	5344	4.39	4082	3.56	15312	2525	227	6.28			
**TAR-13_30**	6493	3042	4.56	1133	2.91	10571	2327	1558	5.27			
**TAR-13_35**	7274	5474	4.91	3987	3.61	4562	2424	1437	4.04			
**TAR-13_39**	11667	6810	4.25	5992	3.41	15247	3447	0*				
**TAR-13_41**	7606	3715	4.83	2785	3.73	17534	5305	3651	4.99			
**TAR-14_1**	12436	9069	5.12	3454	3.34	3009	1705	1356	4.00	148712	146362	145547
**TAR-14_4**	10393	7341	5.40	1013	2.93	8647	5419	3922	5.55	1023027	886676	879402
**TAR-14_5**	4251	3254	3.99	1059	1.86	1974	1064	724	5.86	1020	590	520
**TAR-14_6**	7573	5312	3.93	1976	2.03	9112	4794	3868	2.25	587	414	109
**TAR-14_7**	2038	1265	5.71	586	3.76	6318	3729	2055	6.04			
**TAR-14_10**	6037	4720	3.68	1218	2.50	5809	3158	2412	5.32			
**TAR-14_11**	4899	3453	5.47	614	3.42	4453	2404	1893	4.76			
**TAR-14_12**	7387	5701	4.29	3909	2.54	1892	979	425	4.30			
**MIT-12_7**	5375	869	6.15	412	4.11	21459	1712	988	6.45			
**MIT-12_19**	6007	2342	6.12	1240	4.44	6503	1979	963	5.92			
**ICE-12_3**	5474	2013	5.55	1509	4.50	6383	2333	1602	5.13	9206	1443	4*
**ICE-12_4**	1908	597	6.02	359	4.47	2533	818	444	5.27	18323	6294	540

* removed from analysis due to low sequence numbers

Supplementary Table 9: Algal cell counts, size and biomass

Snow algal cell counts for the Svalbard (SVA), Sweden (TAR) and Greenland (MIT) samples, average diameter of the cells in each sample and inferred cell volume (assuming a spherical cell shape) and biomass (cell counts x volume). No cell counts for the Iceland samples. Large variations in cell counts of individual samples may be derived from possible interferences with inorganic impurities that make cell enumerations in snow samples challenging [3].

Sample ID	Cell counts [mL^{-1}]	Diameter [µm]	Volume [μm^3]	Biomass [$mm^3\ L^{-1}$]
**SVA-13_2**	28125 ± 4419	6.4 ± 2.0	137	4
**SVA-13_4**	15104 ± 2210	12.0 ± 3.4	904	14
**SVA-13_10**	8750 ± 11110	15.8 ± 4.4	2064	18
**SVA-13_20**	32292 ± 24307	n.d.	n.d.	n.d.
**SVA-13_23**	61875 ± 6187	n.d.	n.d.	n.d.
**SVA-13_31**	75000 ± 35355	n.d.	n.d.	n.d.
**SVA-13_33**	6875 ± 2652	12.0 ± 2.5	904	6
**SVA-13_36**	n.d.	12.1 ± 3.4	n.d.	n.d.
**SVA-13_43**	9191 ± 4864	20.0 ± 7.6	4187	38
**SVA-13_48**	23438 ± 6629	n.d	n.d	n.d
**SVA-13_54**	43750 ± 6629	13.0 ± 4.7	1150	50
**SVA-13_65**	27344 ± 16573	18.0 ± 6.3	3052	83
**TAR-13_1**	31250 ± 22097	14.3 ± 4.1	1530	36
**TAR-13_5**	23438 ± 11049	n.d	n.d	n.d
**TAR-13_8**	3438 ± 1326	15.0 ± 5.0	1766	3
**TAR-13_17**	26787	n.d.	n.d.	n.d.
**TAR-13_21**	6757 ± 2389	18.2 ± 3.9	3155	15
**TAR-13_27**	16667 ± 10312	19.0 ± 3.9	3590	36
**TAR-13_28**	n.d.	16.0 ± 2.7	n.d.	n.d.
**TAR-13_32**	29785 ± 7596	13.9 ± 3.1	1405	35
**TAR-13_36**	6875.	17.1 ± 3.1	2617	18
**TAR-13_39**	15625 ± 4419	18.6 ± 5.1	3368	2
**TAR-13_41**	41146 ± 6629	21.5 ± 4.4	5201	14
**TAR-13_42**	31250 ± 5682	12.5 ± 3.0	1022	36
**MIT-12_7**	25800	n.d.	n.d.	n.d.
**MIT-12_19**	5900	n.d.	n.d.	n.d.

n.d. = not determined

Supplementary Table 10: Fatty acid composition

Fatty acid composition of the Svalbard (SVA) and Northern Sweden (TAR) samples as they contained sufficient particulate material for analysis. Fatty acid compounds are reported as percentage of total fatty acids. Most prominent fatty acids are reported as well as total saturated (SFA), total monounsaturated (MUFA) and total polyunsaturated (PUFA) fatty acids. b=branched, A = Alkane.

Although the sample preparation method, i.e. the filtration step, could potentially allow the extraction of fatty acids from non-algal sources, e.g. bacteria, the suite of fatty acids recovered from both Svalbard and Northern Sweden is characteristic of snow algae, with high abundance of monounsaturated and polyunsaturated C18 fatty acids and C16:0 and C16:4 fatty acids[4]. Site TAR-13-1 has the only occurrence of a branched C15 fatty acid, which has not been reported from snow algae but is typical of bacteria, including Sphingobacteria[5]. This is consistent with the dominance of Sphingobacteria OTUs (91.1 % of total bacterial sequences) at this site (**Supplementary** Table 2). The absence of putative bacterial fatty acids at other sites likely reflects lower abundance relative to algal taxa.

Sample ID	C14:0	C15:0	C15 b	C16:0	C16:1	C16:3	C16:4	C18:0	C18:1	C18:2	C18:3	C18:4	C20:0	C21 A	C22:0	C23 A	C24:0	C27 A	SFA	MUFA	PUFA
**SVA-13_2**	0	0	0	25	0	2	12	5	16	9	15	10	3	0	2	0	0	0	34	16	49
**SVA-13_4**	2	0	0	25	1	2	13	3	15	5	20	9	3	0	1	0	0	0	35	16	50
**SVA-13_10**	0	0	0	28	0	0	12	8	2	7	31	9	1	0	0	0	0	0	37	2	59
**SVA-13_20**	0	0	0	23	2	2	15	4	13	7	27	7	0	0	0	0	0	0	27	14	59
**SVA-13_23**	3	0	0	23	2	2	15	3	12	6	27	7	0	0	0	0	0	0	29	14	57
**SVA-13_31**	0	0	0	24	4	2	12	4	15	9	22	9	0	0	0	0	0	0	27	19	53
**SVA-13_33**	0	0	0	23	7	1	6	6	15	10	18	5	2	0	1	1	0	0	32	23	40
**SVA-13_36**	1	0	0	30	2	1	7	5	15	8	14	6	2	1	2	0	0	2	40	17	37
**SVA-13_43**	1	0	0	20	3	2	8	4	17	8	14	3	4	1	2	0	1	0	32	20	36
**SVA-13_48**	0	0	0	20	3	2	12	3	13	10	21	9	0	0	0	0	0	0	24	16	54
**SVA-13_54**	2	0	0	27	1	4	14	2	14	8	18	8	1	0	0	0	0	0	32	15	52
**TAR-13_1**	2	0	1	45	4	5	35	0	0	0	0	0	2	0	1	0	0	0	50	4	40
**TAR-13_5**	3	0	0	26	0	0	19	3	15	1	31	0	0	0	0	0	0	0	32	15	52
**TAR-13_8**	3	0	0	0	3	4	0	6	19	3	37	7	4	0	3	2	2	4	18	22	51
**TAR-13_21**	0	1	0	31	2	2	8	2	13	4	20	13	0	0	1	0	1	0	37	15	48
**TAR-13_27**	0	0	0	42	0	1	7	5	12	4	11	3	4	0	3	1	2	2	56	12	26
**TAR-13_28**	0	0	0	25	1	2	13	5	12	10	16	15	1	0	0	0	0	0	32	13	56
**TAR-13_30**	0	0	0	39	0	0	4	14	8	6	6	4	5	1	3	1	2	2	62	8	19
**TAR-13_32**	0	0	0	22	2	2	16	4	13	7	21	12	0	0	0	0	0	0	26	15	58
**TAR-13_35**	0	0	0	34	0	2	9	17	10	5	14	3	2	0	2	0	0	0	55	10	31
**TAR-13_37**	0	0	0	21	0	2	15	6	12	0	28	14	0	0	0	0	0	0	26	12	60
**TAR-13_39**	0	0	0	26	1	2	13	3	12	7	20	10	1	0	1	0	0	1	31	13	53
**TAR-13_41**	0	0	0	24	2	2	9	7	13	9	18	8	2	0	2	0	0	2	34	16	46
**TAR-13_42**	0	0	0	21	1	3	17	4	13	5	22	12	1	0	0	0	0	0	26	15	58

Supplementary Table 11: Pigment composition

Pigment composition of samples from Svalbard (SVA) and Northern Sweden (TAR) as they contained sufficient particulate material for analysis. Individual pigments were quantified in ug/L and are reported as total chlorophylls, total primary carotenoids and total secondary carotenoids in % of total pigments. Chl a = chlorophyll a, Chl b = chlorophyll b, Neo = Neoxanthin, Vio = Violaxanthin, Ant = Antheraxanthin, Lut = Lutein, Zea = Zeaxanthin, β-car = β-carotene, Ast=Astaxanthin, n.d.= not detected. Although the sample preparation method, i.e. the filtration step, could potentially allow the extraction of pigments from non-algal sources, e.g. bacteria, the suite of pigments recovered from both Svalbard and Northern Sweden is characteristic of snow alga and will be dominated by algal contribution in all cases,

Sample ID	Chl a	Chl b	Neo	Vio	Ant	Lut	Zea	b-car	trans-Ast	cis-Ast	trans- Ast mono esters	cis-Ast mono esters	total Ast di esters	total chloro-phylls [%]	total primary carotenoids [%]	total secondary carotenoids [%]
**SVA-13-2**	3682	3132	215	147	n.d.	1155	n.d.	15	2976	115	62922	6457	1523	8	3	89
**SVA-13-4**	3359	2873	114	175	n.d.	1133	n.d.	81	2664	116	38758	4397	1776	11	4	85
**SVA-13_10**	882	748	25	10	n.d.	351	n.d.	853	1002	15	5164	362	76	17	14	69
**SVA-13_20**	1200	980	100	36	6	573	n.d.	3193	3090	194	17661	1906	2316	7	13	80
**SVA-13_23**	1703	1141	239	290	107	1080	n.d.	1059	4648	372	17305	1916	2779	9	9	82
**SVA-13_31**	327	266	308	468	n.d.	1116	n.d.	51	3927	164	1448	21	178	7	23	69
**SVA-13_33**	534	426	33	26	n.d.	355	n.d.	237	1251	64	3248	201	540	14	10	77
**SVA-13_36**	376	230	93	98	n.d.	155	n.d.	501	1456	86	9142	877	1318	4	6	90
**SVA-13_43**	672	757	56	113	n.d.	577	n.d.	298	868	9	4332	432	1873	14	11	75
**SVA-13_54**	450	507	472	624	n.d.	1525		240	9272	904	5640	546	745	5	14	82
**TAR-13_1**	3253	2712	117	328	0	1499	9	1025	1867	101	28521	3562	4005	8	4	88
**TAR-13_5**	6068	5167	354	670	351	2449	88	957	3546	266	48066	6860	6299	8	4	88
**TAR-13_8**	3084	3212	315	1149	73	2574	14	728	3658	225	21286	2508	6384	9	7	84
**TAR-13_17**	2153	1605	402	721	n.d.	1637	n.d.	1136	3292	122	16014	3063	3949	7	8	85
**TAR-13_21**	1323	1266	315	374	n.d.	1384	n.d.	390	2386	158	13318	1449	2447	7	6	87
**TAR-13_27**	478	373	n.d.	n.d.	n.d.	241	n.d.	50	398	n.d.	3145	286	404	10	3	87
**TAR-13_28**	672	417	597	381	n.d.	563	n.d.	88	1886	115	11182	1113	959	4	5	91
**TAR-13_30**	495	328	0	-31	n.d.	89	n.d.	n.d.	243	n.d.	3035	164	202	11	1	89
**TAR-13_32**	2490	2203	304	823	69	2021	7	459	2078	59	20966	2608	2913	8	6	86
**TAR-13_35**	2319	1578	50	162	n.d.	691	n.d.	402	963	14	10994	1557	2416	11	5	84
**TAR-13_36**	492	310	157	154	n.d.	628	n.d.	107	1599	92	6407	551	981	4	6	90
**TAR-13_37**	1655	1328	47	59	n.d.	638	n.d.	102	1351	57	5628	749	1229	15	5	79
**TAR-13_41**	541	426	747	565	48	1180	n.d.	640	2764	222	8401	884	1766	4	12	85
**TAR-13_42**	7724	8730	1007	1391	111	5052	8	778	5032	205	29339	3554	4411	16	9	75
**TAR-14_1**	1414	632	n.d.	n.d.	n.d.	318	n.d.	n.d.	938	n.d.	21910	2344	639	6	24	71
**TAR-14_4**	369	n.d.	n.d.	n.d.	n.d.		n.d.	n.d.	76	n.d.	693	n.d.	n.d.	32	n.d.	68
**TAR-14_5**	2864	1391	n.d.	n.d.	n.d.	528	22	n.d.	207	n.d.	2418	304	47	55	7	38
**TAR-14_6**	14924	10007	822	752	312	4414	n.d.	n.d.	17256	662	141903	13779	3548	12	4	84
**TAR-14_7**	4458	2533	59	n.d.	29	963	6	n.d.	330	n.d.	4565	391	n.d.	52	9	39
**TAR-14_10**	594	n.d.	n.d.	n.d.	n.d.	49	n.d.	n.d.	238	n.d.	6358	663	133	7	2	90
**TAR-14_11**	332	n.d.	n.d.	n.d.	n.d.		n.d.	n.d.	n.d.	n.d.	102	n.d.	n.d.	76	n.d.	24
**TAR-14_12**	4238	2694	16	25	n.d.	1013	4	n.d.	1751	170	45152	5915	1015	11	2	86

Supplementary Table 12: Aqueous geochemical composition

Aqueous geochemical data for dissolved organic carbon (DOC), nutrients (PO_4^{3-}, NO_3^-, SO_4^{2-}) and trace metals (Al to Zn) in the filtered red snow samples from Svalbard (SVA), Northern Sweden (TAR), Greenland (MIT) and Iceland (ICE).

Sample ID	DOC	PO_4^{3-}	NO_3^-	SO_4^{2-}	Cl^-	Al	Ba	Bi	Ca	Cd	Co	Cr	Cu	Fe	K	Mg	Mn	Na	Ni	Pb	S	Si	Sr	Zn
**SVA-13-2**	31	0.19	1043	<	344	0.4	<	<	100	<	<	<	<	0.5	14	44	1.7	228	<	<	12	<	0.2	0.9
**SVA-13-4**	39	0.14	288	<	381	0.9	0.3	<	94	<	<	<	<	0.9	59	46	1.7	248	<	0.0	14	<	0.3	1.3
**SVA-13_10**	17	0.05	1862	<	242	1.1	0.3	<	64	<	<	0.1	<	2.1	19	12	1.3	139	<	0.0	17	<	0.2	1.2
**SVA-13_20**	41	0.04	7010	160	545	2.1	0.2	<	180	0.0	<	<	<	2.8	56	70	2.0	376	<	0.0	34	<	0.7	0.7
**SVA-13_23**	36	0.08	<	<	332	1.7	0.8	<	955	<	<	0.1	0.2	1.6	65	396	3.1	250	0.1	0.0	48	16	0.9	4.5
**SVA-13_31**	51	0.14	<	<	321	2.0	0.5	<	2020	<	<	<	<	2.0	68	687	3.4	244	0.1	0.0	17	<	2.1	0.6
**SVA-13_33**	18	0.02	895	<	342	1.1	0.5	<	69	<	0.2	<	0.3	1.1	27	12	9.5	243	0.1	0.0	<	15	0.4	0.4
**SVA-13_36**	20	0.05	999	<	354	2.1	0.2	<	34	<	<	<	0.2	4.3	45	15	1.9	259	<	0.2	<	<	0.2	0.7
**SVA-13_43**	60	1.21	<	<	136	1.4	5.6	<	212	<	0.2	<	0.2	2.1	27	65	13.0	90	0.3	0.1	50	<	0.6	0.9
**SVA-13_48**	40	6.08	<	<	297	2.3	6.3	<	639	<	0.1	<	0.1	2.9	64	94	6.1	172	0.2	0.0	30	17	0.9	0.4
**SVA-13_54**	38	0.51	<	<	158	1.3	6.0	<	188	<	<	<	<	1.3	31	53	0.7	97	<	0.0	61	<	0.4	1.0
**SVA-13_65**	39	0.71	<	<	254	0.2	6.7	<	22	<	<	<	<	1.6	20	7	0.5	179	<	0.1	20	<	0.4	0.5
**TAR-13_1**	107	0.26	<	<	102	1.0	<	<	2	<	<	<	<	0.4	47	2	0.1	75	<	<	12	11	<	0.4
**TAR-13_5**	170	0.08	<	<	<	1.1	<	<	2	<	<	<	<	0.3	31	1	0.0	40	<	<	15	12	<	0.2
**TAR-13_8**	198	0.05	<	<	<	1.6	<	<	<	<	<	<	<	2.1	97	4	0.2	42	<	0.0	<	24	<	0.9
**TAR-13_17**	75	0.08	<	<	48	1.3	<	<	5	<	<	<	<	0.9	34	2	0.1	16	<	0.0	<	31	<	0.8
**TAR-13_21**	246	0.10	<	<	173	3.4	0.1	<	14	<	<	<	0.1	3.3	28	3	0.2	132	<	0.0	<	<	<	0.5
**TAR-13_24**	n.d.	n.d.	<	<	75	n.d.	n.d.	n.d.	n.d.	n.d.	n.d.	n.d.	n.d.	n.d.	n.d.	n.d.	n.d.	n.d.	n.d.	n.d.	n.d.	n.d.	n.d.	n.d.
**TAR-13_27**	n.d.	n.d.	157	<	<	0.8	5.4	<	6	<	<	<	<	2.1	12	1	0.1	45	<	0.0	<	29	0.2	0.7
**TAR-13_28**	n.d.	n.d.	<	<	<	n.d.	n.d.	n.d.	n.d.	n.d.	n.d.	n.d.	n.d.	n.d.	n.d.	n.d.	n.d.	n.d.	n.d.	n.d.	n.d.	n.d.	n.d.	n.d.
**TAR-13_30**	n.d.	n.d.	<	<	90	2.6	5.9	<	127	0.2	<	<	0.5	2.7	66	5	0.2	298	0.5	0.1	<	11	0.4	6.8
**TAR-13_32**	n.d.	n.d.	<	<	<	0.4	5.6	<	4	<	<	<	<	0.9	18	2	0.1	22	<	<	<	<	0.2	0.5
**TAR-13_35**	n.d.	n.d.	256	<	<	1.1	6.1	<	12	<	<	<	<	0.3	32	3	0.2	45	<	0.0	<	<	0.2	1.0
**TAR-13_36**	n.d.	n.d.	<	<	<	1.1	6.0	<	7	<	<	<	<	1.2	38	3	0.1	31	<	0.0	<	<	0.3	0.4
**TAR-13_37**	303	0.07	17	147	310	2.6	1.1	<	55	<	<	<	0.4	2.1	52	10	0.3	190	0.2	0.1	<	13	0.2	2.7
**TAR-13_39**	n.d.	n.d.	<	<	83	0.8	7.1	<	2	<	<	<	<	0.5	20	1	0.0	76	<	0.0	<	<	0.2	0.2
**TAR-13_41**	305	3.18	2351	149	554	7.6	1.8	<	191	<	0.1	<	1.3	2.7	303	11	0.9	414	0.7	0.2	67	16	0.5	12.9
**TAR-13_42**	107	0.48	1240	<	<	3.5	0.2	<	43	<	<	<	0.2	1.9	94	5	0.4	44	<	0.0	<	<	0.2	4.8
**MIT-12_7**	n.d.	n.d.	53	393	458	3.0	1.0		19	0	0	0	0	6	212	7	1	134	0	0	n.d.	24	0	1
**MIT-12_19**	n.d.	n.d.	<	<	291	3.0	3.0		18	0	0	0	0	4	29	3	1	148	0	0	n.d.	22	0	3
**ICE-12_3**	n.d.	n.d.	0	0	114	17	7	22	0	0	1	1	17	38	11	0	99	0	-1	0	n.d.	40	0	25
**ICE-12_4**	n.d.	n.d.	0	181	275	4	13	26	0	0	0	0	3	44	13	1	234	0	-3	0	n.d.	483	0	30

All values except DOC and PO_4^{3-} are given in ppb; NO^{3-}, SO_4^{2-} and Cl^- all determined by IC, all others analysed by ICP-MS; limit of detection (LOC,<) for IC: NO_3^- = 96 ppb, Cl^- = 72 ppb, SO_4^{2-} = 121 ppb, LOD's for ICP-MS: Al, Ba, Co, Cr, Cu, Fe, Mg, Ni, Si, Sr, Zn = 0.1 ppb; Bi, Cd, Mn, Pb = 0.01 ppb; Ca, Na = 1 ppb; K,P,S = 10 ppb; n.d. = not determined.

Supplementary Table 13: Particulate composition

Total particulate carbon (TC), total nitrogen (TN), total phosphorus (TP) and total sulphur (TS) (all based on % of dry weight of solid sample) as well as the organic nitrogen and carbon isotope values from the analysed particulates in the Northern Sweden (TAR) and Svalbard (SVA) samples that contained sufficient particulate material for analyses; listed are also the solid C/N (Redfield: 6.6), C/P (Redfield: 106) and N/P (Redfield: 16) ratios calculated from the TC, TN and TP values. Iceland and Greenland samples did not contain enough particulate material for analyses.

Sample ID	Total C [%]	Total N [%]	Total P [%]	Total S [%]	C/N	C/P	N/P	$\delta^{15}N$ [‰]	$\delta^{13}C$ [‰]
**SVA-13-2**	3.69	0.26	0.06	0.16	14.44	59.17	4.10	-8.86	n.d.
**SVA-13-4**	3.05	0.21	0.06	0.11	14.89	51.17	3.44	-6.37	n.d.
**SVA-13_10**	1.45	0.11	0.06	0.18	12.92	23.64	1.83	-6.92	n.d.
**SVA-13_20**	1.87	0.19	0.15	0.07	10.02	12.44	1.24	0.27	-26.06
**SVA-13_23**	7.40	0.45	0.09	0.12	16.57	86.52	5.22	-3.52	-28.42
**SVA-13_31**	8.85	0.50	0.08	0.14	17.70	111.16	6.28	-2.35	-27.80
**SVA-13_33**	2.98	0.21	0.06	0.06	13.93	47.03	3.38	-5.18	-25.00
**SVA-13_36**	6.27	0.36	0.06	0.10	17.59	107.07	6.09	-5.37	n.d.
**SVA-13_43**	2.73	0.15	0.04	0.10	18.70	62.63	3.35	-2.60	-27.95
**SVA-13_48**	4.90	0.23	0.06	0.12	21.06	79.54	3.78	-6.13	-28.18
**SVA-13_54**	17.38	1.02	n.d.	0.12	17.11	n.d.	n.d.	-4.66	-29.43
**SVA-13_65**	4.53	0.27	0.06	0.10	16.51	74.75	4.53	-5.52	-29.23
**TAR-13_1**	4.15	0.25	0.05	0.02	16.59	84.41	5.09	-4.94	-25.01
**TAR-13_5**	30.04	1.84	n.d.	0.11	16.32	n.d.	n.d.	n.d.	-28.73
**TAR-13_8**	12.90	0.78	n.d.	0.06	16.64	n.d.	n.d.	-1.10	n.d.
**TAR-13_17**	19.68	1.11	n.d.	0.10	17.71	n.d.	n.d.	0.37	-26.59
**TAR-13_21**	8.79	0.41	0.04	0.07	21.60	220.17	10.19	-4.19	-21.80
**TAR-13_24**	8.61	0.47	0.05	0.08	18.17	165.77	9.12	-5.11	-25.32
**TAR-13_27**	8.41	0.33	0.08	0.06	25.70	112.09	4.36	-4.76	-27.74
**TAR-13_28**	12.91	0.57	0.07	0.07	22.63	176.06	7.78	-5.49	-24.63
**TAR-13_30**	11.05	0.28	0.04	0.06	39.41	287.09	7.28	-3.29	-24.86
**TAR-13_32**	5.07	0.34	0.07	0.05	15.02	75.07	5.00	-5.96	-26.61
**TAR-13_35**	9.72	0.46	0.07	0.08	21.02	148.70	7.07	-4.54	n.d.
**TAR-13_36**	25.30	0.90	0.07	0.09	28.07	369.73	13.17	-4.95	n.d.
**TAR-13_37**	9.04	0.39	0.07	0.06	23.09	129.79	5.62	-5.40	n.d.
**TAR-13_41**	9.61	0.50	0.03	0.07	19.13	275.76	14.42	-6.15	n.d.
**TAR-13_42**	11.46	0.55	0.06	0.09	20.76	191.61	9.23	-4.73	n.d.

n.d. = not determined

Supplementary Table 14: Mineralogical composition
Geology and mineralogical composition of the Svalbard, Northern Sweden, Greenland and Iceland samples derived from X-ray diffraction analysis.

Location	Geology	Main minerals
**Svalbard**	Metamorphic and sedimentary rocks	quartz, plagioclase, pyroxene, mica, chlorite, muscovite; Austre Brøggerbreen and Feiringbreen: calcite, dolomite
**Northern Sweden**	Metamorphic rocks (gneisses, amphibolites)	quartz, plagioclase, hornblende, minor contributions from biotite, mica and chlorite
**Greenland**	Metamorphic (gneisses) and igneous rocks (gabbro-anorthosite intrusions)	quartz, plagioclase, smectite, mica, hornblende and chlorite
**Iceland**	Igneous rocks (basalt)	quartz, plagioclase, pyroxene, minor contributions from clays, basaltic glass and hematite

Supplementary References

1 Lutz, S., Anesio, A. M., Villar, S. E. J. & Benning, L. G. Variations of algal communities cause darkening of a Greenland glacier. *FEMS microbiology ecology* **89**, 402-414 (2014).

2 Lutz, S., Anesio, A. M., Edwards, A. & Benning, L. G. Microbial diversity on Icelandic glaciers and ice caps. *Frontiers in microbiology* **6**, 307 (2015).

3 Stibal, M. *et al.* Microbial abundance in surface ice on the Greenland Ice Sheet. *Frontiers in microbiology* **6** (2015).

4 Spijkerman, E., Wacker, A., Weithoff, G. & Leya, T. Elemental and fatty acid composition of snow algae in Arctic habitats. *Frontiers in microbiology* **3** (2012).

5 Prasad, S. *et al.* Arcticibacter svalbardensis gen. nov., sp. nov., of the family Sphingobacteriaceae in the phylum Bacteroidetes, isolated from Arctic soil. *International Journal of Systematic and Evolutionary Microbiology* **63**, 1627-1632 (2013).
